# Awareness of being: a computational neurophenomenological model of mindfulness, mind-wandering, and meta-attentional control

**DOI:** 10.1093/nc/niag046

**Published:** 2026-09-05

**Authors:** Hayato Idei, Keisuke Suzuki, Yuichi Yamashita

**Affiliations:** Department of Information Medicine, National Institute of Neuroscience, National Center of Neurology and Psychiatry, 4-1-1 Ogawa-Higashi, Kodaira, Tokyo 187-8502, Japan; Center for Human Nature, Artificial Intelligence, and Neuroscience (CHAIN), Hokkaido University, Kita 12 Nishi 7, Kita-ku, Sapporo, Hokkaido 060-0812, Japan; Department of Information Medicine, National Institute of Neuroscience, National Center of Neurology and Psychiatry, 4-1-1 Ogawa-Higashi, Kodaira, Tokyo 187-8502, Japan

**Keywords:** computational neurophenomenology, mindfulness, mind-wandering, allostasis, interoception, variational recurrent neural network

## Abstract

Mindfulness has established psychological benefits, such as stress reduction and emotional regulation; however, the underlying computational mechanisms, particularly in relation to mind-wandering, remain unclear. This study aimed to present a hierarchical recurrent neural network-based agent model grounded in the free energy principle to explore these processes within the framework of allostasis. The model integrates interoceptive, proprioceptive, and exteroceptive predictive processing, which minimizes the variational free energy throughout past and future contexts. Simulations of the resting state showed that inferred (mentally simulated) homeostatic context modulated attentional shifts between a ‘being’ mode (focused on present interoceptive perception) and a ‘doing’ mode (involving mentally simulated proprioceptive and exteroceptive sensorimotor behaviors). Further, we identified a meta-attentional parameter that controls these shifts by influencing cognitive attitudes toward internally generated beliefs across network modules spanning from the past to the future. By manipulating this parameter, we replicated mindfulness states (‘deep-being’ mode) in which attention remained focused on the present interoceptive state, despite minimal sensory prediction errors. This study offers insights into the computational dynamics of mindfulness and mind-wandering, laying the groundwork for the future exploration of consciousness and pure awareness, which is a state of consciousness devoid of content.

## Introduction

Mindfulness meditation, rooted in Buddhist traditions, is a mental training method that involves focusing on one’s awareness of the present and accepting experiences without evaluation or judgment. Its efficacy has been demonstrated in contemporary times, and it has received significant attention in Western psychology and neuroscience (Kabat-Zinn 1982, Zhang et al. 2021, Bunjak et al. 2022). Mindfulness meditation has been reported to have various psychological and physiological benefits including stress reduction, emotional stability, and enhanced attention. Additionally, neuroscientific research has begun to elucidate its effects on brain structure and function (Chiesa and Serretti 2010, Marchand 2014, Siew and Yu 2023, Yue et al. 2023). However, these studies were primarily based on empirical observations, with specific conscious states induced by mindfulness, and the underlying neural mechanisms remaining unclear.

Computational approaches and modeling have recently gained prominence (Golubickis et al. 2023, Borghesi et al. 2024, Shinagawa and Yamada 2025). Several models based on the free energy principle, which is a unifying theory of brain function, have been proposed. The free energy principle posits that the brain performs information processing, including perception and action, by minimizing the prediction error between its internal models of the world and the actual sensory inputs from the environment, thus providing a unified explanation of perception, learning, and behavior (Friston 2005, Friston 2010, Friston et al. 2010, 2015). A key feature is the integration of perceptual inference, which updates internal states by revising beliefs about the world, and active inference, which interacts with the environment to reduce prediction errors, both of which fall within the framework of minimizing free energy. By leveraging the advantage of the free energy principle to uniformly handle elements such as perception and action, bottom-up sensory information, and top-down beliefs and expectations, which have previously been studied separately, there have been attempts to apply this principle to mindfulness.

Farb et al. (2015) examined the differences between mindfulness and mind-wandering from the perspective of the free energy principle. They proposed that mind-wandering corresponds to a ‘doing mode’, a process of attempting to control sensations and situations based on prior beliefs, which aligns with active inference. However, they proposed that mindfulness corresponds to a ‘being mode’, wherein one distances oneself from thoughts and emotions, accepts sensory inputs as they are, and minimizes the influence of prior beliefs, which aligns with perceptual inference. Manjaly and Iglesias (2020) further explored these modes from the perspectives of ‘decentering’—liberation from self-centered control—and ‘cognitive reactivity’—excessive reactions to incoming stimuli. They suggested that in the ‘being mode’, there was a decreased top-down influence of prior beliefs and increased precision of sensory inputs. Lutz et al. (2019) examined focused attention meditation from the perspective of the free energy principle, considering the top-down attentional focus on breathing to enhance the precision of prediction errors. They interpreted the transition from mindfulness to mind-wandering as a shift in precision weighting from the sensory layers to higher hierarchical cognitive layers. These studies have crucially contributed to the objective analysis of conscious states induced by mindfulness and the elucidation of the underlying information-processing mechanisms. However, computational approaches in previous studies have largely been limited to conceptual descriptions of processes such as precision, prediction errors, and belief updates within the free energy principle without addressing the dynamics of how these variables change during free energy minimization.

Using an agent-based model grounded in the free energy principle (Idei et al. 2025), the present study aimed to investigate how the behaviors, internal states, and neural activities characteristic of mindfulness emerge. Within the framework of the free energy principle, there has been increasing interest in the role of interoception, which governs the maintenance of biological homeostasis, leading to models that incorporate interoceptive sensations and physiological state control (Seth et al. 2011, Seth 2013, Pezzulo et al. 2015, Allen et al. 2022, Tschantz et al. 2022). Similarly, our model integrates interoceptive sensations that perceive an agent’s energy state with exteroceptive and proprioceptive inputs that receive environmental information and generate actions. Furthermore, we introduced the minimal biological assumption that the overall precision of the sensory inputs decreases as the agent’s energy state abnormally declines or increases (Padamsey et al. 2022). Another feature of our model is that the agent modifies its internal state and selects actions to minimize both past and future free energy (Millidge et al. 2021). By developing a multimodal predictive model of the environment through learning, the agent’s focus on the ‘here and now’ versus attention to the distant future and past can be self-organized through the inference of the homeostatic context. Previous studies have demonstrated that modes (attention allocations) naturally emerge within this model, wherein the agent either weighs exteroceptive and proprioceptive inputs to drive the perception-action loop (doing mode) or prioritizes interoceptive sensations to halt actions and conserve energy (being mode) (Idei et al. 2025).

Our model, which explicitly includes interoceptive sensations and minimizes future free energy, offers several advantages over previous models of mindfulness based on the free energy principle. First, although previous models have associated mindfulness with the precision of sensory inputs, many studies have directly and arbitrarily manipulated this precision based on their hypotheses. In contrast, our model allows for automatic adjustment within the process of minimizing the free energy, which naturally emerges through the interaction between the agent and the environment. Second, our model introduces exteroceptive and proprioceptive inputs that drive the perception-action loop based on epistemic motivations, as well as interoceptive sensations involved in maintaining biological homeostasis. By observing which modality becomes dominant, we can identify the ‘being’ and ‘doing’ modes frequently mentioned in computational studies on mindfulness. Third, by introducing a cognitive attitude toward the entirety of internally generated beliefs, we can parametrically manipulate the weighting of (meta-)attention toward the modes, i.e. attention allocations (‘here and now’ versus internally generated beliefs). Based on these three points, our model allows intuitive manipulation of the mindfulness state—where attention is sustained on the ‘here and now’ while reducing mind-wandering—and allows us to analyze an agent’s emergent behaviors and internal states with respect to the balance between sensory information and internal beliefs, as well as the shifts between the ‘being’ and ‘doing’ modes and their meta-attentional control.

In particular, meta-attentional control of the ‘being’ and ‘doing’ modes may offer a deeper perspective on the relationship between mindfulness and mind-wandering. Sustaining attention on present sensory experiences is essential in mindfulness, and this is considered to be achieved by inhibiting mind-wandering (Mrazek et al. 2013, Rahl et al. 2017). Otherwise, during perceptual inference, as prediction errors are minimized, internal beliefs gain a relatively greater influence, leading to spontaneous transitions into mind-wandering. In this sense, mindfulness can be understood as a form of meta-attentional control that regulates the tendency to shift into a mind-wandering state, suggesting that mindfulness operates at a higher level than mind-wandering in the attentional hierarchy. Thus, mindfulness may not simply be a state of ‘being mode’ (perceptual inference) but rather the intentional maintenance of ‘being mode’, which may be more appropriately conceptualized as a ‘deep-being mode’.

In this study, we first describe the agent’s environment and our model based on the free energy principle, followed by the construction and simulation of the agent model. Mindfulness and mind-wandering may be associated with a (meta)attentional balance between incoming sensory information and internally generated beliefs. Considering their relationship with emotional processing, this study aimed to explore the computational mechanisms underlying mindfulness and mind-wandering within the context of interoceptive prediction and allostatic (homeostatic) processing to deepen our understanding of mindfulness and provide new perspectives for the generative mechanisms underlying pure awareness, which is a state of consciousness devoid of content.

## Materials and methods

### Environmental setting

We conducted a simulation experiment with a neural agent to investigate the effects of a cognitive attitude toward the agent’s own internal beliefs on mentally simulated behaviors and spontaneous neural dynamics. The experiment comprised two phases: learning an internal predictive model of a dynamic environment, followed by interoceptive perception and allostatic goal modulation during the resting state.

**Figure 1:**
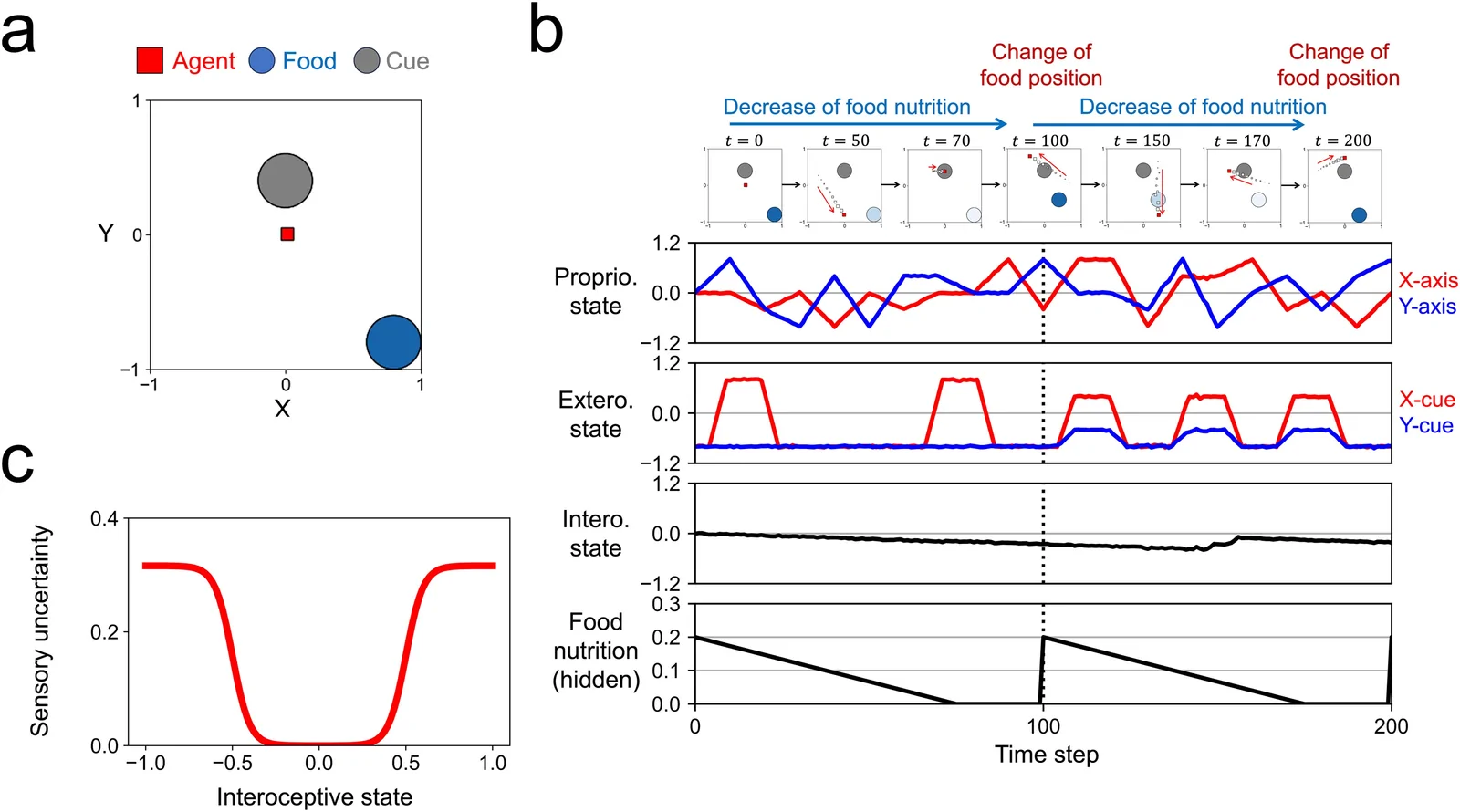
Learning environment. (a) Environmental setup. (b) Time series of multimodal sensations and food nutrition during random-exploration learning. Each sensation takes values between −1.0 and +1.0, corresponding to the range of neural network output. The first 200 steps of learning data (100 000 steps) are shown. (c) The relationship between the interoceptive state and sensory uncertainty (standard deviation).

A learning experiment was conducted to develop an internal predictive model of the agent’s environment. We designed a simulation environment to explore the learning of multimodal predictive models associated with interoceptive, proprioceptive, and exteroceptive sensations, as well as allostatic energy (interoceptive) regulation, in a dynamic environment. In the simulated square space $\left[-\mathrm{1.0,1.0}\right]\times \left[-\mathrm{1.0,1.0}\right]$ (Fig. 1a), food was placed at a random position. When the agent approached it, its energy state increased. The nutritional value and position of the food changed dynamically, as shown in Fig. 1b. A cue placed at a fixed position (0.0, 0.4) temporarily informed the agent of the *x*–*y* coordinate of the current food position if the agent approached it. The agent’s sensations comprised two-dimensional *x*–*y* coordinates of the body position, a two-dimensional cue signal, and a one-dimensional energy state. For convenience, we refer to these sensations as proprioceptive, exteroceptive, and interoceptive.

Additionally, to incorporate the homeostatic context of the agent, we focused on the fact that abnormal interoceptive conditions lead to increased sensory uncertainty (Padamsey et al. 2022), such as dizziness or sluggishness during malnutrition, dehydration, or overhydration. This association between interoceptive conditions and the homeostatic context may be related to increased entropy within the body (Schrödinger 1944, Aoki 1990), in which abnormal interoceptive states may increase the tendency toward disorder. Therefore, sensory (information) entropy or uncertainty is a potential source for the brain to recognize the homeostatic context of the body. This interpretation of homeostasis suggests that the homeostatic context may not only be related to the interoceptive state but also to all aspects of the body, including the sensory organs. We introduced this interpretation of homeostasis into the environmental setting by defining the relationship between the interoceptive state and the standard deviation of sensory noise as the summation of sigmoid functions (Fig. 1c). Additionally, we assumed that movement leads to energy expenditure (a decrease in the interoceptive state). Details regarding the environmental setting have been previously described (Idei et al. 2025). As learning data for our neural network model, we recorded multimodal sensory experiences during random explorations of the environment (Fig. 1b). After learning, we analyzed the mentally simulated behaviors and spontaneous neural dynamics generated by the trained neural agent during the resting state, as described below.

### Computational model

To integrate the neural, computational, and cognitive aspects of mindfulness and mind-wandering within the context of homeostatic processing, we required a computational model characterized by four elements. First, the model must possess the characteristics of a biological neural network, including information processing through synaptic connections and hierarchical network interactions. Second, there should be a predictive (generative) model that represents how multimodal sensory information is altered by internal and external factors in the environment. Third, there must be a computational principle that describes the mechanisms of learning, perceptual inference, and allostatic goal modulation. Finally, it should consider a meta-attentional parameter representing the cognitive attitudes toward internally generated beliefs. As a computational framework that fulfills these requirements, we utilized a multimodal Bayesian homeostatic recurrent neural network model (MBH-RNN) (Idei et al. 2025), which was proposed to explain multimodal predictive processing and allostatic goal modulation based on the free energy principle.

**Figure 2:**
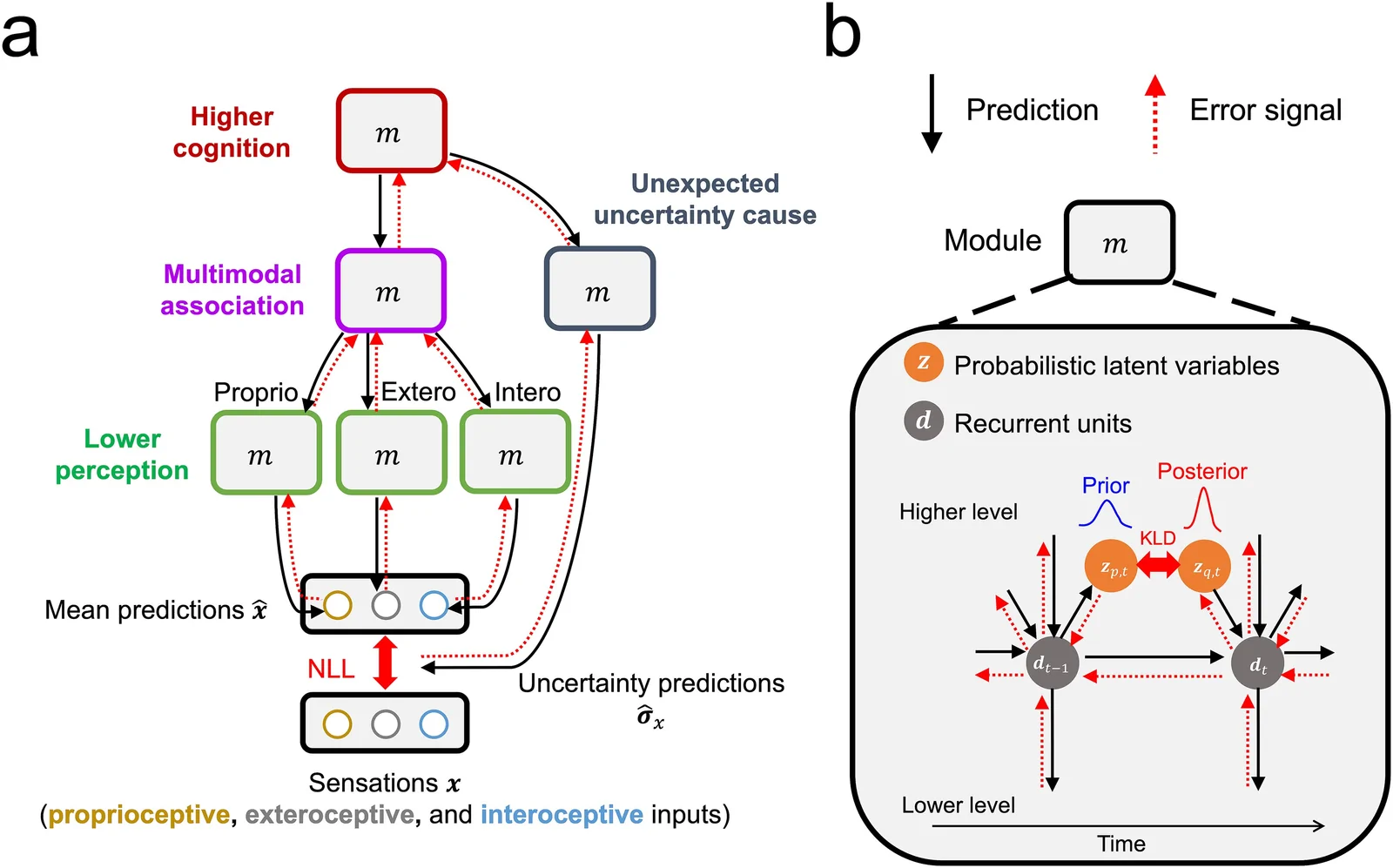
Multimodal Bayesian homeostatic recurrent neural network. (a) Structure and information processing of multimodal Bayesian homeostatic recurrent neural network (MBH-RNN). (b) Information processing inside each module. Prior and posterior distributions of latent variables are represented as single Gaussian distributions for clarity; however, each of them is a multivariate Gaussian distribution. NLL: negative log-likelihood. KLD: Kullback–Leibler divergence.

Previous neuroimaging studies have suggested that allostatic interoceptive processing involves a large-scale brain network composed of core regions of salience and default mode networks (Kleckner et al. 2017). Our previous study (Idei et al. 2025) suggested a rough correspondence between the functional architectures of the allostatic-interoceptive network in the brain and the MBH-RNN, which comprises sensorimotor and homeostatic modules (Fig. 2). The sensorimotor modules of the MBH-RNN include lower perceptual modules (distributed for interoception, proprioception, and exteroception) and a multimodal associative module. They hierarchically represent interoceptive information and its associations with exteroceptive and proprioceptive information. In contrast, the homeostatic modules include the higher cognitive module and the unexpected-uncertainty-cause module, which represent different types of uncertainty information associated with the homeostatic context of the agent. The higher cognitive module integrates multimodal information with sensory uncertainty information and thus represents the uncertainty information that can be predicted from internal and external environmental states. Contrastingly, the unexpected-uncertainty-cause module is associated with the perception of unexpected causes of sensory uncertainty that are independent of (or could not be expected by) the internal and external environmental state. The MBH-RNN allowed the examination of how the balance between the perception of the present interoceptive state (being mode) and the sensorimotor loop, driven by epistemic motivation and involving exteroceptive and proprioceptive predictions (doing mode), is regulated through the inference of the homeostatic context. We previously showed that a neural agent controlled by the MBH-RNN exhibited allostatic interoceptive (energy) regulation in the environment through autonomous switching between sensory-focused resting (being mode) and goal-directed moving (doing mode) (Idei et al. 2025).

**Figure 3:**
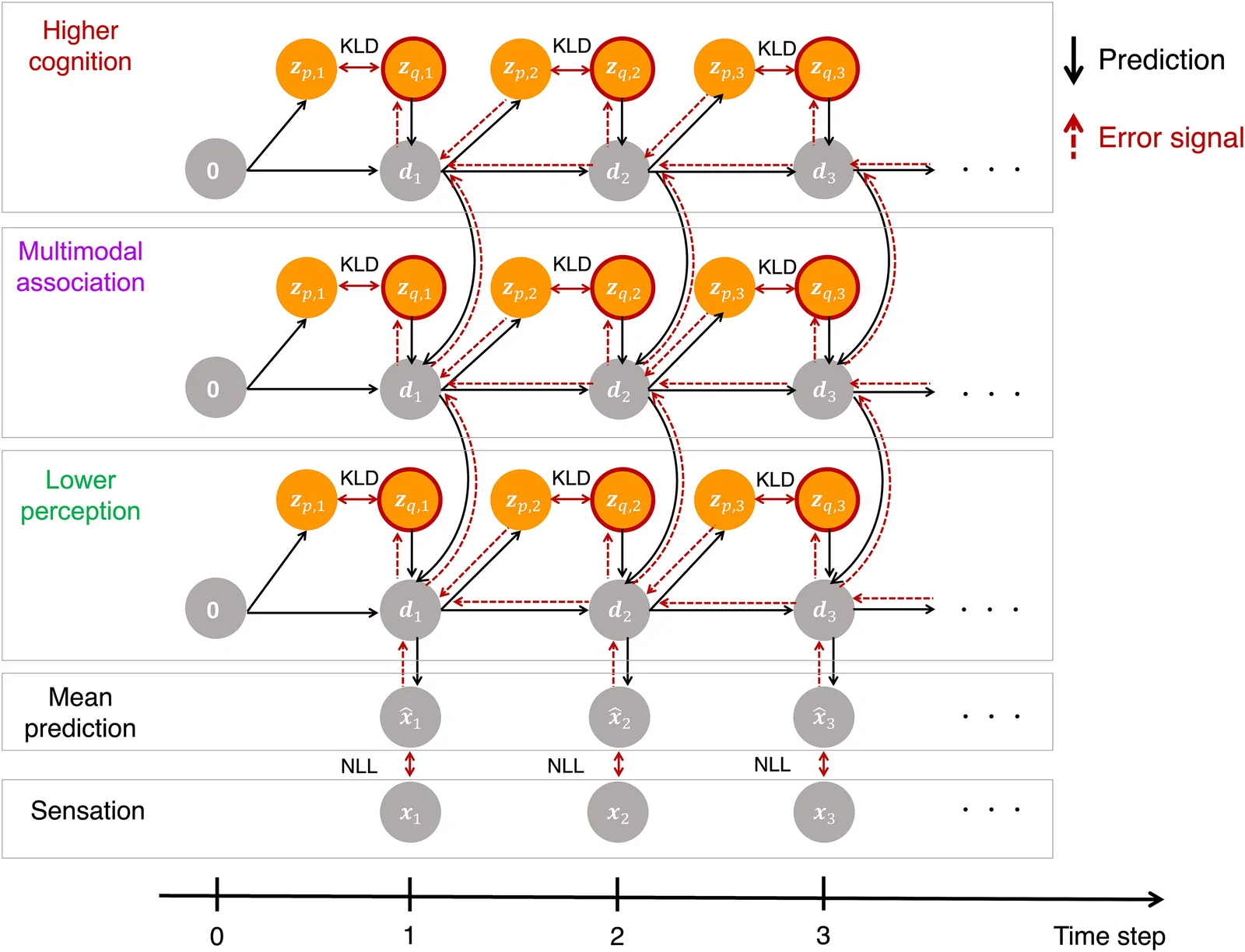
Temporal processing of hierarchical modules in the MBH-RNN. In the learning process, the MBH-RNN updates the time-invariant synaptic weights $\mathrm{\omega}$ and the posterior distributions ${z}_{q,1:T}$ in all modules via minimization of variational free energy over the time length of the learning data *T*. For simplicity, the distributed structures of the lower-perceptual modules, as well as the unexpected-uncertainty-cause module and sensory-uncertainty prediction, are omitted.

Each module of the MBH-RNN is based on a predictive-coding-inspired variational RNN (Idei et al. 2022), in which probabilistic latent variables represent an agent’s internal belief regarding the cause of sensation and uncertainty (Figs 2 and 3). Each latent variable has a prior distribution $\boldsymbol{p}\left({\boldsymbol{z}}_t^{(m)}|{\boldsymbol{d}}_{t-1}^{(m)}\right)$ and a posterior distribution $\boldsymbol{q}\left({\boldsymbol{z}}_t^{(m)}|{\boldsymbol{e}}_{t:T}\right)$, corresponding to the estimated hidden causes before and after observing sensations, respectively (*m*: module, ${\boldsymbol{d}}_{t-1}^{(m)}$: previous states of RNN units in the same module, ${\boldsymbol{e}}_{t:T}$: backpropagated errors generated in the current and future timesteps, *T*: the last timestep of the time window). Based on the posterior states, the MBH-RNN generates predictions about sensations in a top-down manner, where the RNN units transform latent states into sensory predictions via synaptic connections that represent the dynamic relationships between sensations and their causes. The MBH-RNN learns by updating time-invariant connection weights $\boldsymbol{\omega}$ and the sequential posterior distributions ${\boldsymbol{z}}_{q,1:T}$ over the length of learning data in all modules via variational free energy (VFE) minimization. The mathematical details of the prediction generation and learning process are provided in the Supplementary material.

**Figure 4:**
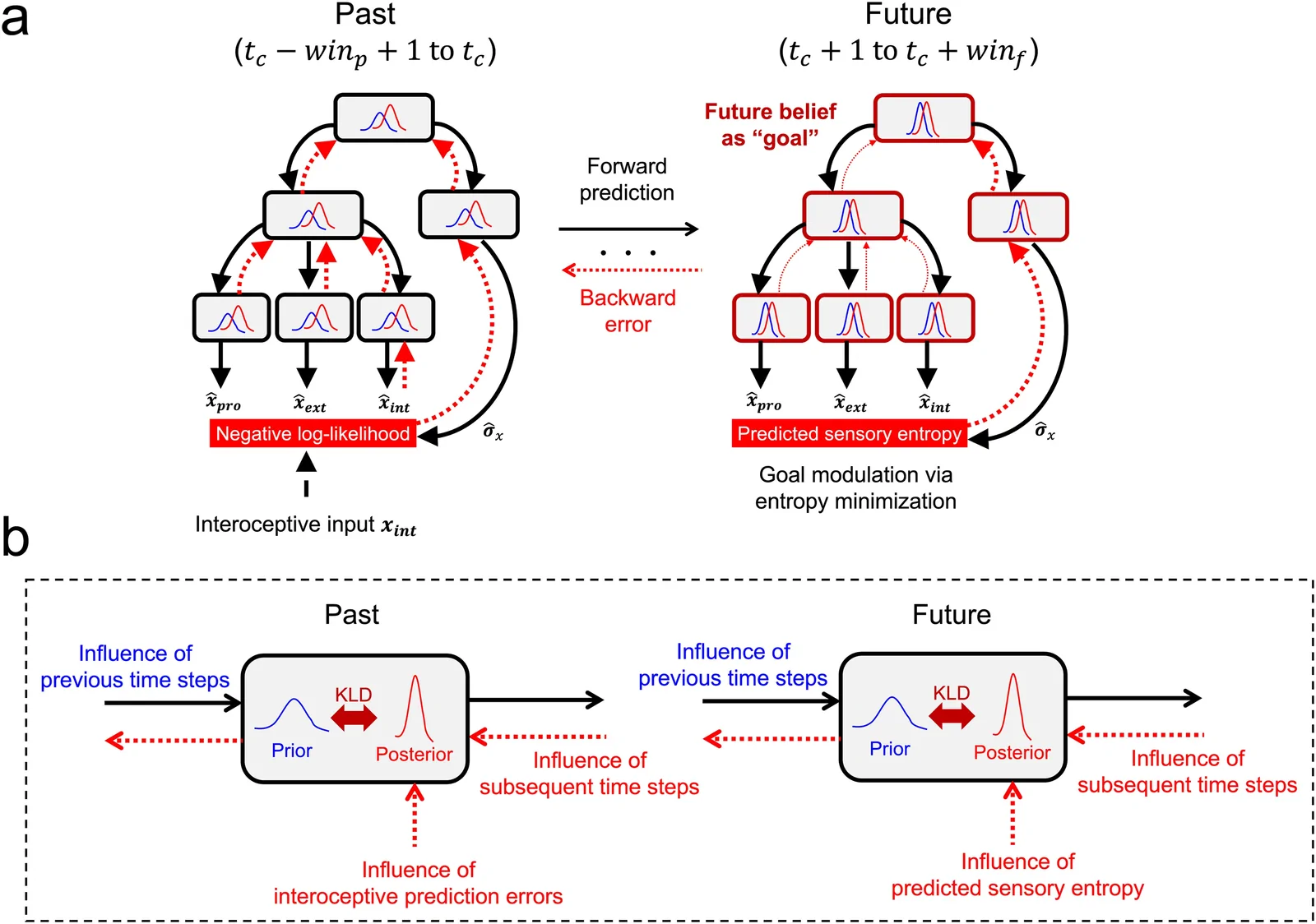
Online perceptual inference and allostatic goal modulation during the resting state. (a) Predictive processing from past to future during the resting state experiment. ${t}_c$, ${win}_p$, and ${win}_f$ indicate the current network timestep, the length of the past time window, and the length of the future time window, respectively. (b) A simplified diagram that illustrates how posterior distributions at a certain timestep are influenced by sensory information and beliefs at previous and subsequent timesteps.

Here, we briefly explain the process of online perceptual inference and goal modulation in the resting state after learning. Perceptual inference and goal modulation were implemented by considering posterior updates based on VFE minimization from past to future. The synaptic weights were fixed after completing the learning phase. At the current network timestep ${t}_c$, the MBH-RNN updates the posteriors within the timesteps from ${t}_c-{win}_p+1$ to ${t}_c+{win}_f$ (${win}_p$ and ${win}_f$are the lengths of the past and future time windows, respectively) (Fig. 4). The cost function in the past time window ${F}_{past}$ is the accumulated VFE of the past (VFEP), which is calculated based on the observed sensations ${\boldsymbol{x}}_{t_c-{win}_p+1:{t}_c}$.


(1) \begin{eqnarray*} {F}_{past}=\sum_{t={t}_c-{win}_p+1}^{t_c}{\mathrm{VFEP}}_t \end{eqnarray*}

(2) \begin{eqnarray*} {\displaystyle \begin{array}{c}{\mathrm{VFEP}}_{\mathrm{t}}\!=\!\!\underset{\mathrm{Negative}\ \mathrm{accuracy}}{\underbrace{-\ln p\left({\boldsymbol{x}}_t|{\boldsymbol{d}}_t\right)}}\!\!+\!W\sum_m\underset{\mathrm{Complexity}}{\underbrace{D_{KL}\left[q\left({\boldsymbol{z}}_t^{(m)}|{\boldsymbol{e}}_{t:{t}_c+{win}_f}\right)\left\Vert p\right({\boldsymbol{z}}_t^{(m)}|{\boldsymbol{d}}_{t-1}^{(m)}\Big)\right]}}\end{array}} \end{eqnarray*}

**Figure 5:**
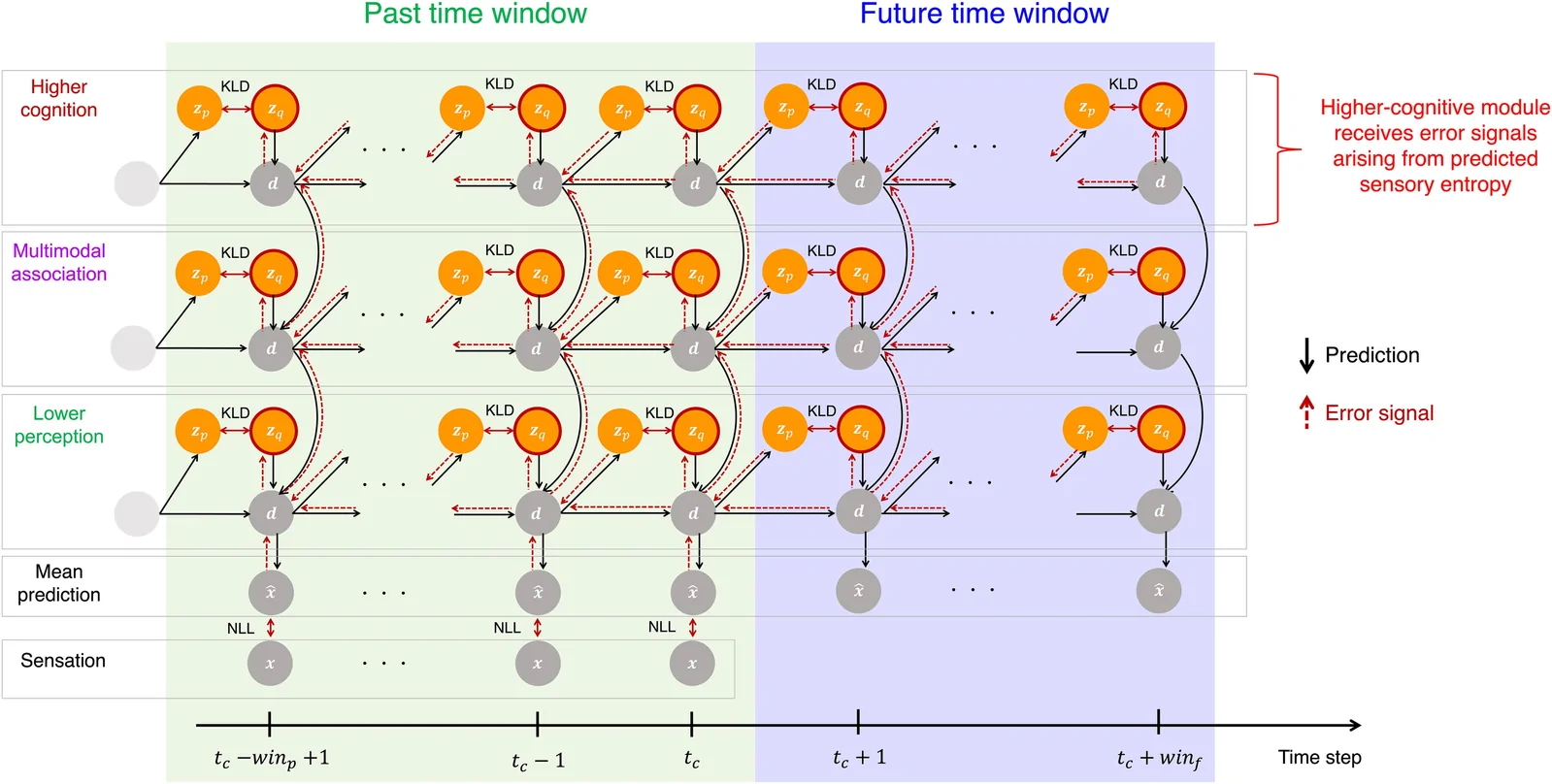
Temporal processing of online perceptual inference and allostatic goal modulation. At the current network timestep ${t}_c$, the MBH-RNN updates the posterior distributions ${z}_{q,{t}_c-{win}_p+1:{t}_c+{win}_f}$ in all modules by minimizing the variational free energy from the past to the future. The synaptic weights were fixed after the learning process was complete. For simplicity, the distributed structure of the lower-perceptual modules, as well as the unexpected uncertainty cause module and sensory uncertainty prediction, are omitted.

The first term (negative accuracy term) of the VFE describes the negative log-likelihood (NLL) or precision-weighted sensory prediction error. The second term (complexity term) of the VFE describes the Kullback–Leibler divergence between the posteriors and priors. In addition, we introduced a meta-attentional parameter called meta-prior *W*, which determines the balance between the negative accuracy and complexity terms (Ahmadi and Tani 2019) (explained below).

However, for future predictive processing, we extended the VFE to the future time window while considering future sensations as unknown variables. Specifically, we derived a VFE form of the future (VFEF) by evaluating VFE under the expectation of predictions about future sensations. This changes the NLL term of the VFE to the predicted conditional (Shannon) entropy of sensation (Idei et al. 2025). VFEF is distinct from the concept of expected free energy, which was derived from a different mathematical basis to explain action planning while balancing exploration and exploitation within the free energy principle (Friston et al. 2015, Millidge et al. 2021). Instead, this VFEF derivation allows for a unified formulation of perceptual and active inferences within VFE minimization (Idei et al. 2025). The cost function in the future time window ${F}_{future}$ is the VFEF accumulated over the future time window.


(3) \begin{eqnarray*} {\displaystyle \begin{array}{c}{F}_{future}=\sum_{t={t}_c+1}^{t_c+{win}_f}{\mathrm{VFEF}}_t\end{array}} \end{eqnarray*}

(4) \begin{eqnarray*} {\displaystyle \begin{array}{c}{\mathrm{VFEF}}_t=\underset{\mathrm{Predicted}\ \mathrm{sensory}\ \mathrm{entropy}}{\underbrace{\mathcal{H}\left[p\left({\boldsymbol{x}}_t|{\boldsymbol{d}}_t\right)\right]}}\\+W\sum_m\underset{\mathrm{Predicted}\ \mathrm{complexity}}{\underbrace{D_{KL}\left[q\left({\boldsymbol{z}}_t^{(m)}|{\boldsymbol{e}}_{t:{t}_c+{win}_f}\right)\left\Vert p\right({\boldsymbol{z}}_t^{(m)}|{\boldsymbol{d}}_{t-1}^{(m)}\Big)\right]}}\end{array}} \end{eqnarray*}

This derivation of the VFEF allows the formalization of allostatic processing to avoid an increase in sensory entropy (uncertainty). See the Supplementary material and our previous study (Idei et al. 2025) for more mathematical details regarding the derivations of VFEP and VFEF.

Taken together, the MBH-RNN, with fixed synaptic weights, repeats prediction generations and updates of posterior distributions ${\boldsymbol{z}}_{q,{t}_c-{win}_p+1:{t}_c+{win}_f}$ within timesteps from ${t}_c-{win}_p+1$ to ${t}_c+{win}_f$ to minimize the accumulated free energy from past to future ${F}_{allostasis}$ (Fig. 5).


(5) \begin{eqnarray*} {\displaystyle \begin{array}{c}{F}_{allostasis}={F}_{past}+{F}_{future}\end{array}} \end{eqnarray*}

Through the minimization of ${F}_{allostasis}$, the MBH-RNN simultaneously performs perceptual inference and goal modulation, in which we regard future beliefs as a future ‘goal’ that is adapted toward minimizing predicted future sensory entropy. Notably, this ‘goal’ is not an externally given objective; instead, it is an internal objective resulting from the process of minimizing free energy. In online processing, posterior distributions at a certain timestep are influenced by sensory prediction errors, predicted future sensory entropy, and internal beliefs at previous and subsequent timesteps. The influences of previous and subsequent beliefs arise from the complexity terms of VFEP and VFEF, which are weighted by meta-priors. Here, the meta-prior can be considered a cognitive attitude toward the entirety of the internally generated probabilistic beliefs across network modules, spanning from the past to the future (rather than focusing on specific beliefs).

### Experimental setting

To investigate the effects of cognitive attitudes toward internally generated beliefs on mentally simulated behaviors and neural dynamics, we analyzed online interoceptive inference and allostatic goal modulation in the resting state. The experiment consisted of learning and resting state phases. The learning phase of the MBH-RNN applied multimodal sensory data acquired through random exploration of the environment for 100 food nutrition cycles (10 000 timesteps) (Fig. 1b). As aforementioned, the MBH-RNN learns to reconstruct the target data by updating the synaptic weights and posterior distributions (see the Supplementary material for mathematical details). We trained ten MBH-RNNs with different initial synaptic weights.

Subsequently, the trained MBH-RNNs experimented on interoceptive inference and goal modulation in the resting state. Specifically, to clearly analyze spontaneous network behavior through mental simulation, we assumed that the interoceptive state was constantly 0.0 (${x}_{t,\mathit{\operatorname{int}}}=0.0$ for all *t*), assuming a meditative state with a calm interoceptive condition rather than an emergency state such as severe hunger. Additionally, when implementing a mental simulation of sensorimotor behavior, exteroceptive and proprioceptive prediction errors did not influence posterior updates by setting ${\boldsymbol{x}}_{t,\mathit{\operatorname{ext}}}={\hat{\boldsymbol{x}}}_{t,\mathit{\operatorname{ext}}}$ and ${\boldsymbol{x}}_{t, pro}={\hat{\boldsymbol{x}}}_{t, pro}$ for all *t* (see Supplementary material). In this setting, the MBH-RNN repeats prediction generations and posterior updates within timesteps from ${t}_c-{win}_p+1$ to ${t}_c+{win}_f$ 200 times online based on the minimization of ${F}_{allostasis}$. As mentioned earlier, the synaptic weights were fixed during this phase, following the completion of the learning phase.

To simulate alterations in cognitive attitude (meta-attention), we manipulated the meta-prior *W*, which modulates the weight of the complexity terms in VFEP and VFEF. Specifically, as the meta-prior increases, the influence of the entirety of internally generated beliefs on posterior updates is weighted higher than that of the sensory information. Therefore, the meta-prior *W* modulates meta-level attention to internal probabilistic beliefs (estimated distributions, not just their mean states) relative to incoming sensory information. Specifically, small and large meta-priors characterize sensory- and belief-focused cognitive attitudes, respectively. Therefore, we hypothesized that meta-prior modulation may be a potential target for simulating the characteristics of mindfulness and mind-wandering. We analyzed the effects of meta-prior modulations $W=\left\{0.1,0.2,0.5,1.0,2.0,5.0,10\right\}$ on network behavior to explore the computational mechanisms of mindfulness and mind-wandering (*W=1.0* is the baseline).

One trial of the resting state is 2000 timesteps ($1\le{t}_c\le \mathrm{2000}$), with 10 trials being performed by each of the 10 trained MBH-RNNs in each meta-prior setting. In our previous study (Idei et al. 2025), the lengths of the past and future time windows were ${win}_p=10\ \left(\mathrm{or}\ {win}_p={t}_c+1\ if\ {t}_c<10\right)$ and ${win}_f=200$, respectively. See the Supplementary material for details on the settings of the other hyperparameters of the MBH-RNN.

### Statistical analysis

One-way repeated-measures analysis of variance (ANOVA) was used for all statistical analyses, followed by multiple comparisons adjusted using the Shaffer method. All statistical tests were two-tailed, and the significance level was set at *P<.05*. No statistical methods were used to determine the sample size. Considering the high reproducibility of the computational simulation, we set the minimum sample size as statistically testable (10 samples). Data analyses were conducted using the R software (version 3.3.2).

## Results

### Modulations of cognitive attitude affected the calmness of the network behavior

**Figure 6:**
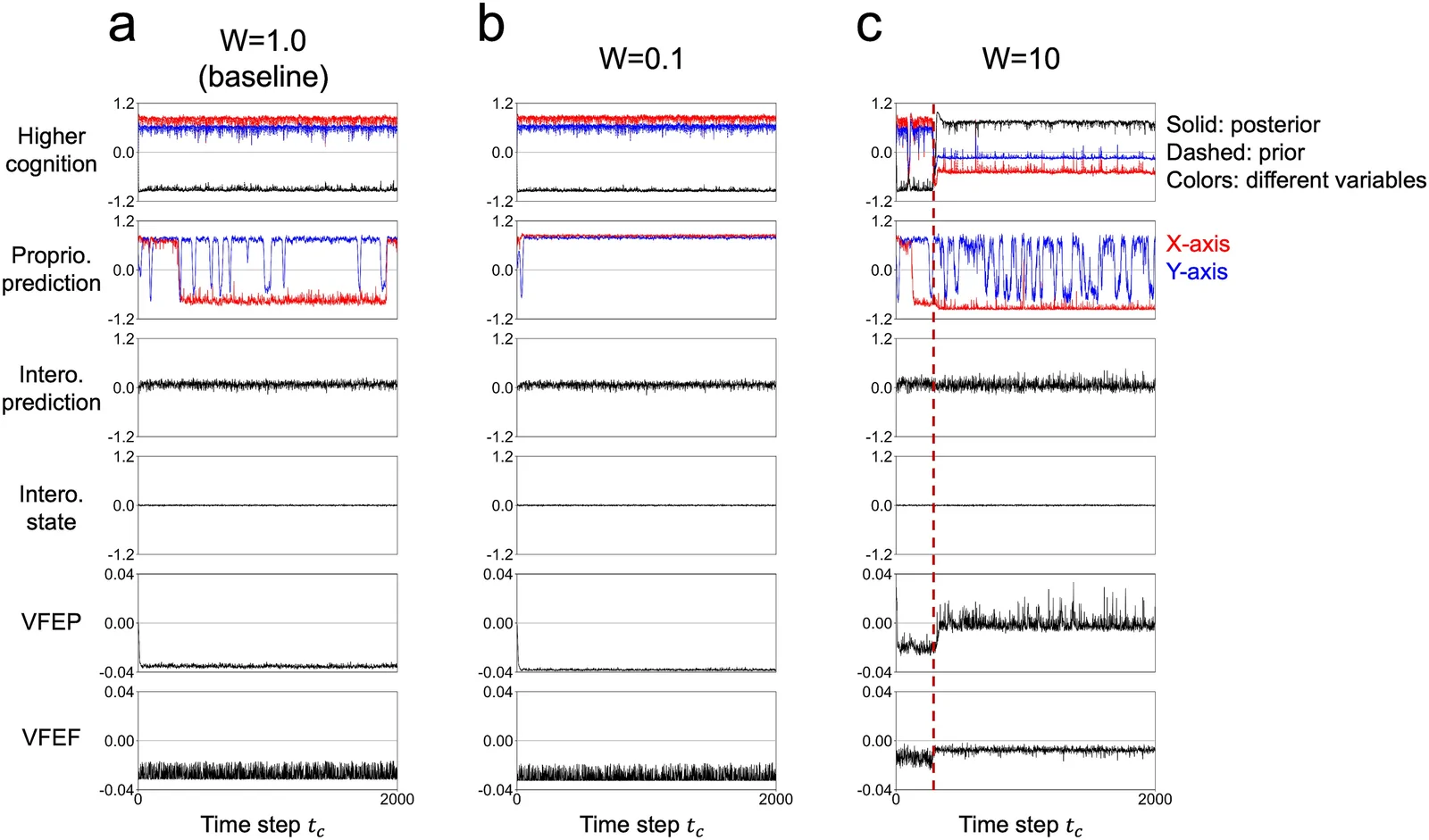
Alterations of general network behavior induced by modulations of meta-prior. (a) An example of the ‘being’ mode under baseline condition (*W=1.0*). (b) An example of the ‘deep-being’ mode under the small meta-prior condition (*W=0.1*). (c) An example of transitions between the ‘being’ and ‘doing’ modes under the large meta-prior condition (*W=10*). The vertical line indicates when VFEF was spontaneously generated. Higher cognition: mean states of latent variables in the higher cognitive module; Proprio. prediction: sensory predictions about proprioceptive inputs; Intero. prediction: sensory prediction about the interoceptive input; Intero. state: interoceptive input; VFEP: variational free energy of the past averaged over the length of the past time window; VFEF: variational free energy of the future averaged over the length of the future time window.

First, we qualitatively analyzed the generated time series of the latent states and sensory predictions during the resting state. Figure 6a shows a case (an example) of the baseline conditions (*W=1.0*). The network successfully predicted a constant interoceptive state, with low levels of VFEP and VFEF. The mean states of the latent variables in the higher cognitive module remained consistent throughout the trials. These observations indicated that the network recognized the calm homeostatic context and remained in the ‘being’ mode (perceptual inference minimizing sensory prediction errors). Additionally, the proprioceptive predictions of the network moved intermittently and occasionally remained unchanged. We referred to these mentally simulated states of movement and stillness as ‘wandering’ and ‘resting’.

Decreased and increased meta-priors affected mentally simulated behaviors and neural dynamics differently. As shown in Fig. 6b, a small meta-prior (*W=0.1*) induces a reduction in the changes in proprioceptive predictions. This suggests that a sensory-focused cognitive attitude with a small meta-prior can increase resting and decrease wandering, deepening the ‘being’ mode. In contrast, in the large meta-prior condition (*W=10*), a large VFEF was spontaneously generated, as indicated by the brown vertical line in Fig. 6c. The increased VFEF changed the latent states in the higher cognitive module, leading to increased fluctuations in proprioceptive predictions and a large VFEP. This suggests that a belief-focused cognitive attitude can induce internally caused modulations in the recognition of the homeostatic context, yielding the ‘doing’ mode. Taken together, modulations of meta-priors affect the calmness of spontaneous network behavior.

**Figure 7:**
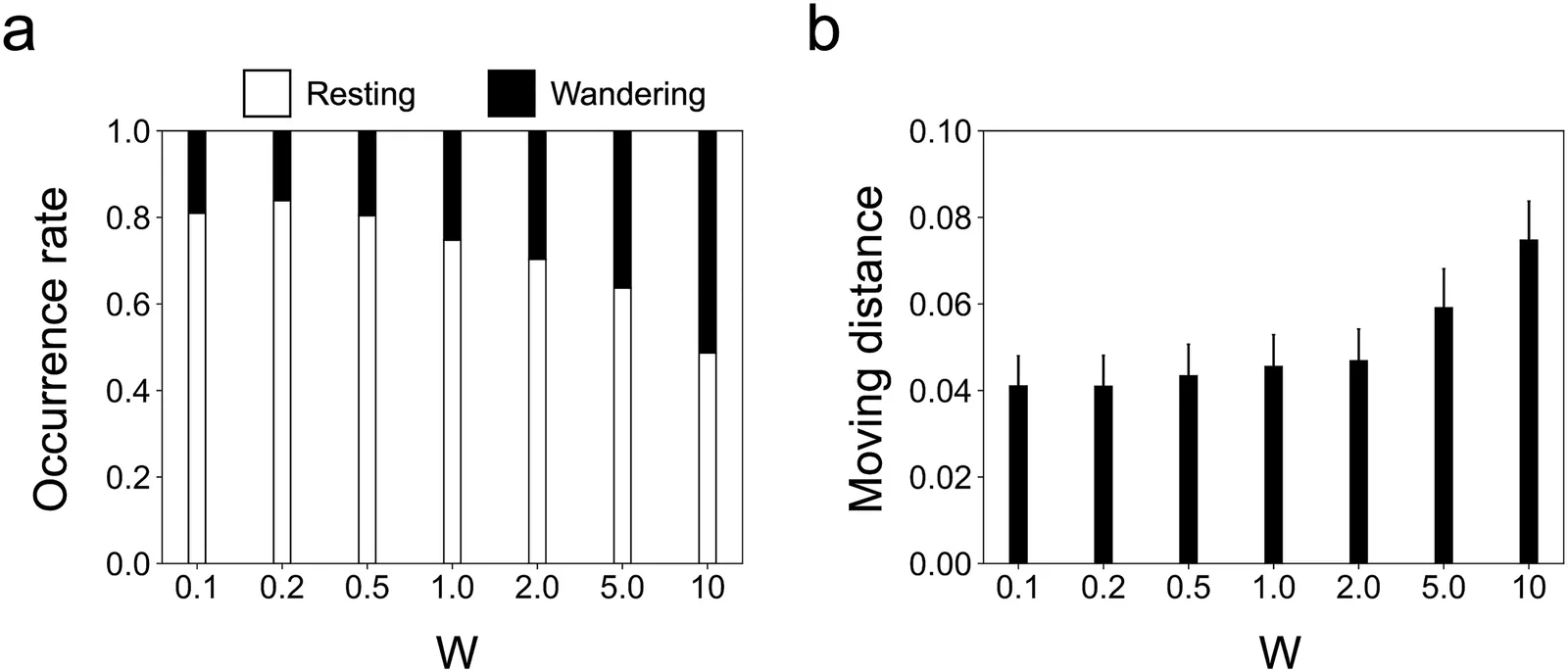
Alterations of mentally simulated behavior induced by modulations of meta-prior. (a) Occurrence rates of resting and wandering. (b) Moving distance per timestep, calculated as changes in proprioceptive predictions. These were analyzed using data after the end of the posterior updating process throughout the trial. Error bars indicate the standard error.

Based on the qualitative results, we quantitatively evaluated the mentally simulated behaviors. To calculate the rates of resting and wandering occurrence, fluctuations in proprioceptive predictions were quantitatively assessed at every 50 timesteps and classified into resting and wandering. Specifically, a proprioceptive sequence of a 50-timestep bin is classified as ‘resting’ if the differences between the proprioceptive predictions at any timestep within the bin and those at the first timestep are smaller than 0.5. Specifically, if the predicted position of the agent remained stationary within the 50-timestep bin, the state is regarded as ‘resting’, while other cases are classified as ‘wandering’. The analysis was conducted for all 10 trials and 10 trained networks. The results summarized in Fig. 7a show that resting decreased (wandering increased) as the meta-prior increased, which is consistent with the qualitative analysis. Resting occurrence rates were compared between the three groups of the small meta-prior condition (*W=0.1,0.2,0.5*), baseline condition (*W=1.0*), and large meta-prior condition (*W=2.0,5.0,10*) using one-way repeated-measures ANOVA. In the small and large meta-prior conditions, the rates of resting occurrence were averaged over three meta-prior settings: *W=0.1,0.2,0.5* and *W=2.0,5.0,10*, respectively. ANOVA revealed a significant between-group difference (*P=.0012*, *n=10*). Additionally, *post hoc* multiple comparisons adjusted using the Shaffer method revealed that the rate of resting occurrence was significantly reduced in the large meta-prior condition than in the baseline (*P=.0012*) and small meta-prior conditions (*P=.0078*), while there was a mild difference between the baseline and small meta-prior conditions (*P=.16*).

The effects of meta-prior modulation on mentally simulated behavior were also reflected in the mentally simulated moving distance, which was calculated as the change in proprioceptive predictions per timestep (Fig. 7b). Specifically, the moving distance per timestep increases under the large meta-prior condition. One-way repeated-measures ANOVA revealed a significant difference between the small meta-prior, baseline, and large meta-prior conditions (*P=.0014*, *n=10*). Furthermore, *post hoc* multiple comparisons using the Shaffer method revealed that the moving distance was significantly increased in the large meta-prior condition compared with the baseline (*P=.0058*) and small meta-prior conditions (*P=.0089*), whereas there was no significant difference between the baseline and small meta-prior conditions (*P=.31*). These results suggest that an increase in meta-prior disrupted the calmness of mentally simulated behavior by increasing transitions between the ‘being’ and ‘doing’ modes, whereas a decrease in meta-prior mildly deepened the ‘being’ mode. We confirmed that the deep-being mode (increase in resting) was not induced by alterations in the length of the past or future time windows; however, a decreased length of the past time window led to a decrease in resting (increase in wandering) (Supplementary Fig. S1).

### Different network dynamics were involved in sensory-focused and belief-focused cognitive attitude

**Figure 8:**
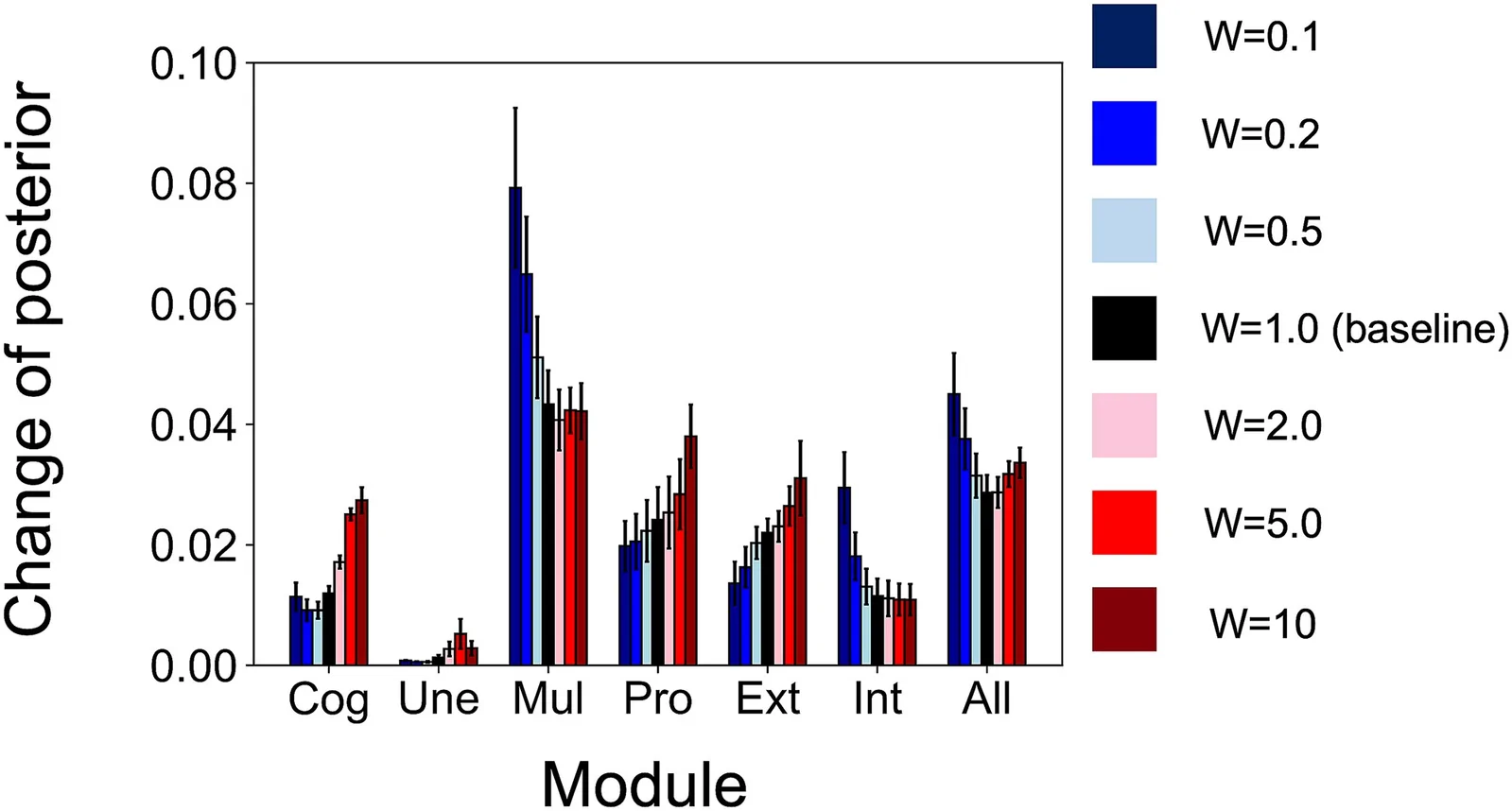
Neural-level alterations induced by meta-prior modulations. Changes in the mean states of posteriors per timestep in each module. These were analyzed using data obtained after the end of the posterior updating process throughout the trial. Cog: Higher cognitive module; Une: unexpected-uncertainty-cause module; Mul: multimodal-associative module; Pro: proprioceptive module; Ext: exteroceptive module; Int: interoceptive module; All: all modules. Error bars indicate standard error.

Next, we investigated the dynamics of the latent states underlying the alterations in mentally simulated behavior caused by the modulation of meta-priors. We calculated the changes in the mean states of the posteriors in each network module, which can be regarded as a measure of the extent to which the corresponding network module is involved in perception and sensorimotor predictions. The values were averaged over all latent variables, 2000 timesteps, and 10 trials in each module. As shown in Fig. 8, the effects of the meta-prior modulation depended on the homeostatic, multimodal associative, and lower-perceptual modules. Specifically, in the multimodal associative and interoceptive modules, changes in the posteriors increased as the meta-prior decreased. This suggests that a sensory-focused cognitive attitude with small meta-priors increased engagement in perceiving the interoceptive state and its associations with other sensory modalities, which may underlie the ‘deep-being’ mode. In contrast, as the meta-prior increased, the proprioceptive and exteroceptive modules showed increased changes in their posterior states, which may reflect an increase in the ‘doing’ mode. Additionally, a belief-focused cognitive attitude with large meta-priors induced fluctuating beliefs about the homeostatic context represented by the homeostatic modules, especially the higher cognitive module, which may increase posterior changes in the proprioceptive and exteroceptive modules (Fig. 6). Taken together, homeostatic modules, especially the higher cognitive module, switch attention allocation between the perception of the current interoceptive state (being mode) and the sensorimotor loop involving exteroceptive and proprioceptive processing (doing mode).

### Dynamics of free energy characterized shifts between the ‘being’ and ‘doing’ modes

Finally, we investigated the effects of meta-prior modulation on the level and variability of free energy, which characterized the posterior updates, and thus may underlie general network behavior, including mentally simulated behavior and neural dynamics. First, we compared the levels and variabilities of VFEP and VFEF among the small meta-prior, baseline, and large meta-prior conditions. VFEP and VFEF levels were calculated as averages over the length of the past and future time windows, respectively. Moreover, VFEP and VFEF levels calculated at each network timestep were averaged over 2000 timesteps and 10 trials. Variabilities of the VFEs (VFEP and VFEF) were calculated by averaging the absolute differences between the VFEs and their median values over 2000 timesteps and 10 trials. The median VFE values were calculated using the time series generated by each network with a specific meta-prior setting.

**Figure 9:**
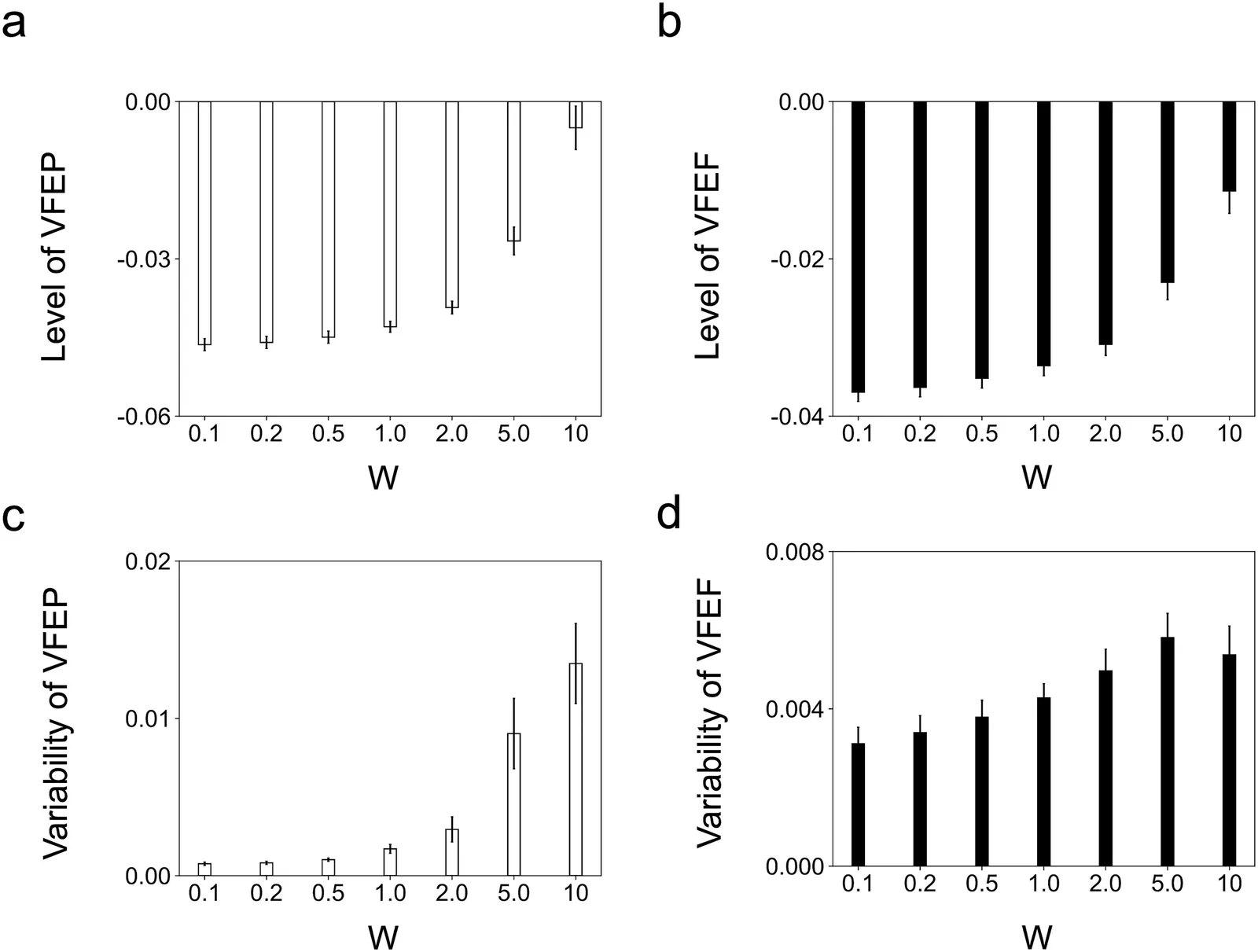
Level and variability of free energy altered by modulations of meta-prior. (a) The level of variational free energy of the past (VFEP). (b) The level of variational free energy of the future (VFEF). (c) The variability of VFEP was calculated as the average of absolute differences from the median value. (d) The variability of VFEF was calculated as the average of absolute differences from the median value. Error bars indicate the standard error.

As summarized in Fig. 9, the levels and variabilities of VFEP and VFEF increase with increases in the meta-prior. One-way repeated-measures ANOVA revealed significant differences in VFEP and VFEF levels between the small meta-prior, baseline, and large meta-prior conditions (*P<.0001* for both, *n=10*). *Post hoc* multiple comparisons adjusted using the Shaffer method revealed significant differences in VFEP levels in all pairs of the three conditions (*P<.0001* for baseline versus large meta-prior, *P<.0001* for small meta-prior versus large meta-prior, and *P=.0003* for baseline versus small meta-prior). Moreover, there were significant differences in the VFEF levels in all pairs of the three conditions (all *P<.0001*). These results show that VFEP and VFEF levels increased as the meta-prior increased.

Furthermore, one-way repeated-measures ANOVA revealed significant differences in the variabilities of VFEP and VFEF among the three conditions (*P<.0001* for VFEP and *P=.009* for VFEF, *n=10*). For VFEP, *post hoc* multiple comparisons adjusted using the Shaffer method revealed significant differences in VFEP variabilities in all pairs of the three conditions (*P=.0014* for baseline versus large meta-prior, *P=.0018* for small meta-prior versus large meta-prior, and *P=.026* for baseline versus small meta-prior). For VFEF, there were significant differences between the baseline and small meta-prior conditions (*P=.0039*), as well as between the small and large meta-prior conditions (*P=.028*). There was a mild difference in the variability of VFEF between the baseline and large meta-prior conditions (*P=.082*). These results confirmed that both VFEP and VFEF fluctuated as the meta-prior increased. These significant impacts on VFE dynamics may characterize the deepening of the ‘being’ mode by small meta-priors as well as the increased transitions between the ‘being’ and ‘doing’ modes due to large meta-priors.

**Figure 10:**
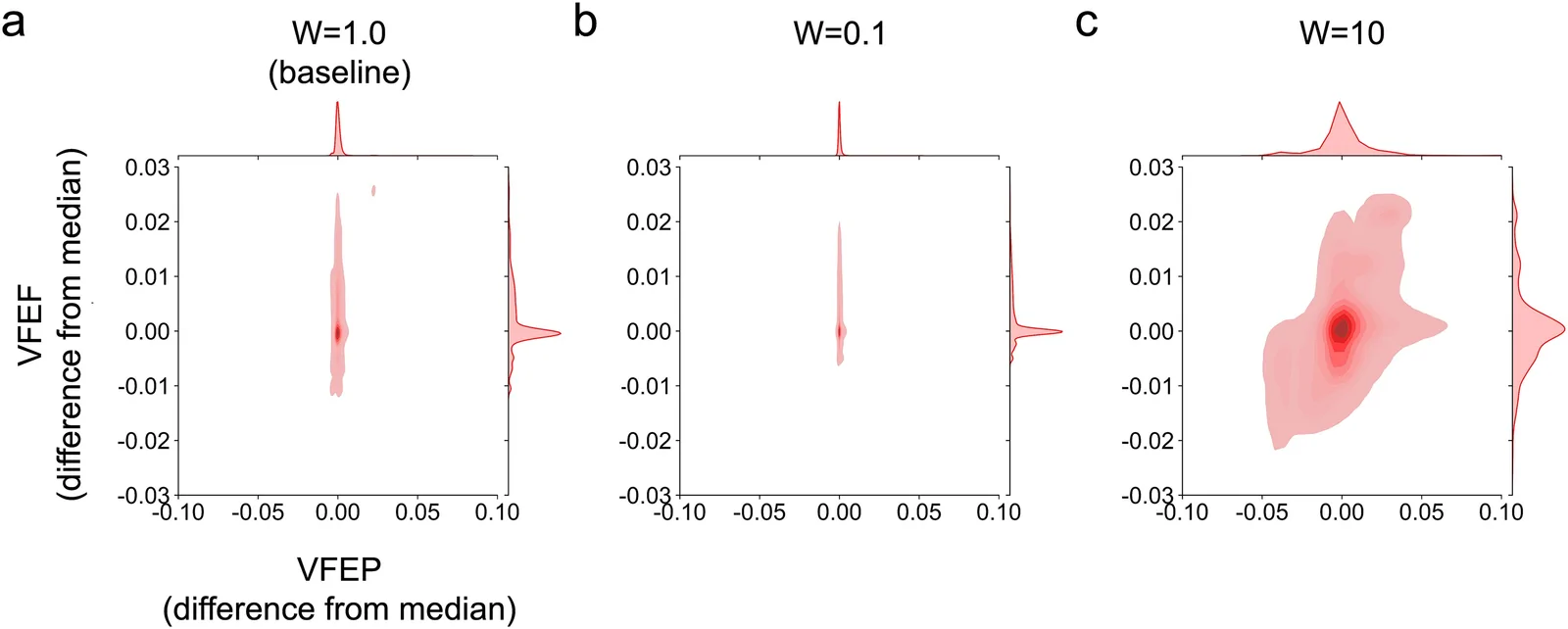
Distribution of free energy altered by modulations of meta-prior. (a) The relationship between variational free energy of the past (VFEP) and variational free energy of the future (VFEF) under the baseline condition (*W=1.0*), presented as a two-dimensional distribution. VFEs are shown as differences from the median values. (b) The VFE distribution under the small meta-prior condition (*W=0.1*). (c) The VFE distribution under the large meta-prior condition (*W=10*). Error bars indicate the standard error.

To extract the qualitative characteristics of VFEP and VFEF, we display the relationship between VFEP and VFEF as a two-dimensional distribution (Fig. 10). The distribution was created by plotting pairs of VFEP and VFEF for all 2000 timesteps, 10 trials, and 10 networks in each meta-prior setting. For clarity, VFEP and VFEF were plotted as differences from the median values. As shown in Fig. 10b, the network with a small meta-prior remained in a narrow state within the two-dimensional VFE space compared to the baseline condition. This indicates that under a sensory-focused cognitive attitude, the network behavior converged into a stable free energy state, with low VFEP and VFEF levels and variabilities (Fig. 9). In contrast, the distributions of VFEP and VFEF were widespread in the large meta-prior condition (Fig. 10c). Specifically, the distribution of the VFEF exhibited multimodality, with several smaller peaks occurring in addition to the largest peak at higher VFEF values. This may be associated with the spontaneous generation of a large VFEF (Fig. 6c). These findings suggest that in a belief-focused cognitive attitude with a large meta-prior, network behavior can fluctuate among multiple free energy states, even if the interoceptive state is calm. Taken together, the dynamics of the free energy state may characterize the network transitions between the ‘being’ and ‘doing’ modes.

## Discussion

This study applied the MBH-RNN to describe the computational mechanisms underlying mindfulness and mind-wandering based on cognitive attitudes toward one’s internal model of the world. This study emphasizes the advantages of MBH-RNN as a hierarchical generative model that is foundational to discussing cognitive phenomena, including mindfulness and mind-wandering. First, it learns to infer the dynamically changing precision (inverse of the standard deviation) of each sensory piece of information and hierarchical hidden causes (environmental states) via VFE minimization. Previous Bayesian models have proposed that mindfulness and mind-wandering can be explained by adjustments in the precision-weighting of sensory information and beliefs; however, precision weighting has been set manually or discussed in abstract descriptions (Lutz et al. 2019, Manjaly and Iglesias 2020, Sandved-Smith et al. 2021). In our model, the precision weighting of each piece of information was epigenetically self-organized through learning as part of the hierarchical probabilistic generative model. Second, the MBH-RNN incorporates predictive processing from the past to the future; further, it integrates perceptual inference and (allostatic) goal modulation within VFE minimization. Third, multimodal predictive processing allows consideration of the modulation of attentional balance among interoception, proprioception, and exteroception through the inference of the homeostatic context. A neural network model that integrates the concepts of network hierarchy, temporal depth, multimodality, and homeostasis may allow the exploration of various cognitive phenomena associated with attention allocation (mode switching) and meta-attention (cognitive attitude), beyond the generative modeling of external environments. Our results demonstrate that the behavioral patterns and neural activity characteristics of mindfulness spontaneously emerged within the model.

Several assumptions were introduced in the current resting-state simulations to isolate the computational mechanisms bridging allostatic inference and mindfulness (mind-wandering). First, exteroceptive and proprioceptive prediction errors were set to zero during online inference, allowing the network to freely generate exteroceptive and proprioceptive predictions without being constrained by incoming sensory information. This setting enabled us to examine spontaneous internally generated sensorimotor predictions and to characterize network dynamics in terms of wandering and resting states. Second, the interoceptive input was generally clamped to 0, corresponding to a calm interoceptive condition. This manipulation enabled us to examine spontaneous belief generation and mode switching while controlling for changes in interoceptive states themselves. In other words, our aim was to investigate how internally generated beliefs and free-energy dynamics shape mindfulness- and mind-wandering-like processes even in the absence of substantial changes in bodily states. Although these assumptions should be regarded as methodological simplifications introduced to isolate the target phenomena within a computational framework, they also limit the ecological validity of the simulations. In particular, mindfulness is often characterized as an acceptance of present-moment experience, which encompasses not only interoceptive sensations but also exteroceptive and proprioceptive experiences. Therefore, future studies should extend the current framework to more realistic environmental settings in which interoceptive, exteroceptive, and proprioceptive signals continuously interact with internally generated beliefs. Such extensions may help clarify both the common and modality-specific characteristics of mindfulness and mind-wandering, as well as the role of multimodal interactions in shaping these phenomena.

Our simulation study suggests that the ‘being’ (perceptual inference) and ‘doing’ modes (active inference) can emerge as a characteristic attentional balance between the perception of the present interoceptive state and the mentally simulated sensorimotor loop involving exteroceptive and proprioceptive predictions. Specifically, the ‘being’ mode was observed as small interoceptive prediction errors in which the homeostatic context during the resting state was appropriately recognized. Conversely, the ‘doing’ mode was observed as an increase in mentally simulated movements (wandering) due to inappropriate inferences about the homeostatic context. Each mode was characterized by a specific free energy state within the space of VFEP and VFEF, in which the ‘being’ and ‘doing’ modes corresponded to small and large VFEs, respectively. Notably, the homeostatic modules, especially the higher cognitive module, were involved in switching between the two modes, depending on the inferred homeostatic context.

Furthermore, by introducing a meta-prior, we demonstrated that the shifts between ‘being’ and ‘doing’ modes can be controlled by modulations of cognitive attitudes toward the entirety of internally generated beliefs across network modules spanning from the past to the future. Consequently, a small meta-prior allowed sustained focus on the interoceptive state, even with a greatly reduced sensory prediction error, where the influences of internally generated beliefs could become dominant. During the deep-being mode, the functions of the multimodal-associative and interoceptive modules became dominant compared to the other modules. In contrast, a large meta-prior induced an increase in the transitions between the ‘being’ and ‘doing’ modes, even in the calm interoceptive state, in which the proprioceptive and exteroceptive modules increased their activation (changes in beliefs) during the doing mode. The different properties of the shifts between the two modes under small and large meta-prior conditions are reflected in the dynamics of the free energy states. Accordingly, meta-priors can be considered meta-attention to the ongoing cognitive mode (attention allocation). Our findings may explain mindfulness as a meta-attentional strategy for suppressing the tendency to diverge from the present sensory experience and reflect on past or future events, thereby clarifying its role in reducing mind-wandering (doing mode). These results provide insights into the computational mechanisms underlying attention allocation and its regulation involving large-scale brain networks, including the default mode, salience, and sensorimotor networks, which exhibit characteristic activities associated with mindfulness and mind-wandering (Brewer et al. 2011, Tang et al. 2015, Kucyi et al. 2024).

Finally, our findings may provide insights into ‘pure awareness’—that is, ‘awareness of being aware’—often reported by experienced meditators. Studies on consciousness have considered mindfulness meditation a crucial tool for investigating transformations of conscious states. Specifically, pure awareness as referenced in Eastern religious traditions such as Tibetan Buddhism represents a domain that remains to be elucidated through Western scientific approaches. Pure awareness refers to the state of directly experiencing consciousness without specific objects or content (Metzinger 2020). This minimal and most fundamental form of conscious experience, which is stripped of self-awareness and perceptions of time and space, holds significant importance for approaching the essence of consciousness and its foundational roots by eliminating extraneous elements from complex conscious phenomena and solely focusing on essential factors (Metzinger 2020, Metzinger 2024). However, scientific investigation of such extreme states of consciousness remains challenging, and phenomenological investigations into pure awareness have only just begun (Alcaraz-Sánchez et al. 2022, Alcaraz-Sánchez 2023, Metzinger 2024, Alcaraz-Sánchez 2025).

From a computational perspective, pure awareness may correspond to a higher-order form of meta-attention, namely an awareness of awareness itself (Metzinger 2024). In our model, the meta-prior is interpreted as a form of meta-attention to ongoing cognitive modes, such as being mode and doing mode. In this sense, the meta-prior supports awareness of the currently active mode of cognition. Extending this interpretation, we suggest that a further level of meta-attention directed toward the meta-prior itself may correspond to an even higher-order form of awareness, namely an awareness of being aware. Such awareness may correspond to hidden meta-awareness states within a deep active inference framework (Sandved-Smith 2024). At this level, awareness is no longer restricted to monitoring the active cognitive mode. Rather, it may reflect the emergence of a perspective that transcends any particular cognitive mode. Decoupled from any single mode of cognition, such awareness instead encompasses the system’s capacity for awareness itself, spanning the full range of possible cognitive modes and the transitions available to it. Understood this way, meta-awareness may involve not only particular experiences or modes, but the system as a whole—its capacity to generate, monitor, and regulate different modes of experience. Because its object becomes the system’s own generative and regulatory processes, such a perspective naturally introduces self-referentiality and epistemic openness characteristic of meta-awareness. Notably, the higher-level cognitive module of our model can predict sensory uncertainty through the unexpected-uncertainty-cause module, which processes uncertainties that cannot be predicted from either internal or environmental states and can drive behavior aimed at resolving unexpected sources of uncertainty (Idei et al. 2025). Although this mechanism was not examined in the current simulations, a reduction in the meta-prior may enhance openness to unpredictable events in the external environment through the unexpected-uncertainty-cause module. Such a process may capture important phenomenological aspects of pure awareness; however, further investigation is required.

Such a higher-order form of awareness may also have implications for recent active inference accounts of consciousness, particularly the Beautiful Loop Theory (Laukkonen et al. 2025). In their account, awareness emerges through ‘epistemic depth’, which refers to the clarity or intensity of knowing itself and the degree to which a system recursively models its own capacity for knowing. More specifically, awareness is proposed to arise when a world model recursively models itself and thereby comes to contain knowledge of its own capacity for cognition. This account emphasizes a recursive form of self-modeling that extends beyond awareness of specific contents toward awareness of the system’s own cognitive capacities. From the perspective of our model, meta-attention directed toward the meta-prior may provide one possible computational basis for the emergence of such epistemic depth. Specifically, awareness of ongoing cognitive modes may support the emergence of a higher-order perspective directed toward the system’s own capacity to generate, monitor, and regulate different modes of experience. Although this connection remains speculative, it suggests that the emergence of meta-attention toward the meta-prior may be a potential computational ingredient in the development of epistemic depth and meta-awareness. An important direction for future research will be to clarify how recursive world models, through ongoing interactions with internal and external environments, may give rise to meta-attention toward the meta-prior, and how such processes may contribute to the emergence of epistemic depth and pure awareness.

Our agent model integrates the neural, computational, and cognitive (attentional) aspects of mindfulness and mind-wandering. Therefore, the proposed mechanisms may be empirically tested by investigating the relationships among various aspects of mindfulness and mind-wandering, including brain activity and phenomenological analyses. Specifically, at the neural level, our analyses of neural dynamics predict that the ‘being’ and ‘doing’ modes would be dissociated by differences in neural activities in brain regions involved in interoceptive perception and multimodal associations, such as the insula ((Bud) Craig 2009, Tang et al. 2015, Wang et al. 2019), or proprioceptive and exteroceptive predictions, such as sensorimotor cortices (Mckeown et al. 2020, Kucyi et al. 2024). In addition, the functionality of homeostatic modules of the MBH-RNN has been identified in homeostatically relevant regions involved in cognitive control and emotional processing, including the salience network, prefrontal cortex, and amygdala (Monosov 2017, Juechems et al. 2019, Seeley 2019). At the cognitive (phenomenological) level, mindfulness may be associated with meta-attention toward an ongoing mode (attention allocation). Accordingly, mindfulness may require attenuation of ‘entire’ internal beliefs across network modules, spanning from past to future (attenuation of a specific belief would not guarantee the success of mindfulness). Furthermore, our findings suggest that the importance of attention to interoception in mindfulness training may be related to reduced inappropriate inferences about the homeostatic context and the resulting negative emotions, such as anxiety, even in the absence of issues with the interoceptive state due to mind-wandering (Feruglio et al. 2021). Furthermore, future studies using computational frameworks, such as model-fitting techniques and individualized (embodied) brain models (Idei and Yamashita 2024, Mindlin et al. 2024, Takahashi et al. 2025), may help extract the dynamic associations among the neural, computational, and cognitive aspects of mindfulness and mind-wandering.

## Supplementary Material

Supplementary_material_niag046

## Data Availability

All data are available in the manuscript and supplementary material. Computer code for the MBH-RNN was written using C++ and is available at https://github.com/h-idei/mbhrnn.git.
