# Supplementary material for: Awareness of being: a computational neurophenomenological model of mindfulness, mind-wandering, and meta-attentional control
Source: Neurosci Conscious. 2026 Sep 5;2026(1):niag046. doi: 10.1093/nc/niag046 (PMC13545998; doi:10.1093/nc/niag046)
Supplement: Supplementary file 1 [file supplementary_material_niag046.docx]

**Supplementary material for**

**Awareness of Being: A Computational Neurophenomenological Model of Mindfulness, Mind-wandering, and Meta-attentional Control**

Hayato Idei^a,*,1^, Keisuke Suzuki^b,*^, and Yuichi Yamashita^c,*^

a. Department of Information Medicine, National Institute of Neuroscience, National Center of Neurology and Psychiatry, Tokyo 187-8502, Japan. Email: [idei@ncnp.go.jp](mailto:idei@ncnp.go.jp)

b. Center for Human Nature, Artificial Intelligence, and Neuroscience (CHAIN), Hokkaido University, Hokkaido, 060-0812, Japan. Email: [ksk@chain.hokudai.ac.jp](mailto:ksk@chain.hokudai.ac.jp)

c. Department of Information Medicine, National Institute of Neuroscience, National Center of Neurology and Psychiatry, Tokyo 187-8502, Japan. Email: [yamay@ncnp.go.jp](mailto:yamay@ncnp.go.jp)

*These authors are joint corresponding authors on this work.

^1^ Lead contact

**A.1. Mathematical details of the neural network model**

***A.1.1. Dynamic prediction generation***

The RNN unit of the MBH-RNN is a conventional firing-rate model. The internal state $h_{t,i}$ and output $d_{t,i}$ of the $i$th RNN unit in each network module at timestep $t$ ($t\geq1$) is calculated as follows:

$$h_{t,i}= \left\{ \begin{aligned} \frac{1}{\tau}\left( \sum_{j\in I_{\text{Cd}}} \omega_{ij}d_{t-1,j} + \sum_{j\in I_{\text{Cz}}} \omega_{ij}z_{t,j}+ b_{i} \right)+\left( 1- \frac{1}{\tau} \right)h_{t-1,i} \left( i\in I_{\text{Cd}} \right) \\ \frac{1}{\tau}\left( \sum_{j\in I_{\text{Ud}}} \omega_{ij}d_{t-1,j} + \sum_{j\in I_{\text{Uz}}} \omega_{ij}z_{t,j}+ \sum_{j\in I_{\text{Cd}}} \omega_{ij}d_{t,j}+ b_{i} \right)+\left( 1- \frac{1}{\tau} \right)h_{t-1,i} (i\in I_{\text{Ud}}) \\ \frac{1}{\tau}\left( \sum_{j\in I_{\text{Ad}}} \omega_{ij}d_{t-1,j} + \sum_{j\in I_{\text{Az}}} \omega_{ij}z_{t,j}+ \sum_{j\in I_{\text{Cd}}} \omega_{ij}d_{t,j}+ b_{i} \right)+\left( 1- \frac{1}{\tau} \right)h_{t-1,i} (i\in I_{\text{Ad}}) \\ \frac{1}{\tau}\left( \sum_{j\in I_{\text{Pd}}} \omega_{ij}d_{t-1,j} + \sum_{j\in I_{\text{Pz}}} \omega_{ij}z_{t,j}+ \sum_{j\in I_{\text{Ad}}} \omega_{ij}d_{t,j}+ b_{i} \right)+\left( 1- \frac{1}{\tau} \right)h_{t-1,i} \left( i\in I_{\text{Pd}} \right) \\ \frac{1}{\tau}\left( \sum_{j\in I_{\text{Ed}}} \omega_{ij}d_{t-1,j} + \sum_{j\in I_{\text{Ez}}} \omega_{ij}z_{t,j}+ \sum_{j\in I_{\text{Ad}}} \omega_{ij}d_{t,j}+ b_{i} \right)+\left( 1- \frac{1}{\tau} \right)h_{t-1,i} \left( i\in I_{\text{Ed}} \right) \\ \frac{1}{\tau}\left( \sum_{j\in I_{\text{Id}}} \omega_{ij}d_{t-1,j} + \sum_{j\in I_{\text{Iz}}} \omega_{ij}z_{t,j}+ \sum_{j\in I_{\text{Ad}}} \omega_{ij}d_{t,j}+ b_{i} \right)+\left( 1- \frac{1}{\tau} \right)h_{t-1,i} \left( i\in I_{\text{Id}} \right) \\ \#(A.1) \end{aligned} \right.$$

$$\begin{aligned} d_{t,i}=\tanh\left( h_{t,i} \right) \left( i\in I_{\text{Cd}},I_{\text{Ud}},I_{\text{Ad}},{{I_{\text{Pd}}, I}_{\text{Ed}}I}_{\text{Id}} \right)\#\left( A.2 \right) \end{aligned}$$

where $I_{\text{Cd}}$, $I_{\text{Ud}}$, $I_{\text{Ad}}$, $I_{\text{Pd}}$, $I_{\text{Ed}}$, and $I_{\text{Id}}$, are the index sets of the RNN units in the higher cognitive, unexpected uncertainty cause, multimodal associative, proprioceptive, exteroceptive, and interoceptive modules, respectively. $I_{\text{Cz}}$, $I_{\text{Uz}}$, $I_{\text{Az}}$, $I_{\text{Pz}}$, $I_{\text{Ez}}$, and $I_{\text{Iz}}$ are the index sets of probabilistic latent variables in the higher cognitive, unexpected uncertainty cause, multimodal-associative, proprioceptive, exteroceptive, and interoceptive modules, respectively. $\omega_{ij}$ is the weight of the synaptic connection from the $j$th unit to the $i$th unit; $z_{t,j}$ is the output of $j$th latent variables at timestep $t$; $\tau$ is the time constant of the RNN unit; and $b_{i}$ is the bias of the $i$th RNN unit. An RNN unit with a small and large time constant $\tau$ tends to rapidly and slowly change its activity, respectively. We set the initial internal states of the RNN units $h_{0,i}$ ($i\in I_{\text{Cd}},I_{\text{Ud}},I_{\text{Ad}},{{I_{\text{Pd}}, I}_{\text{Ed}}, I}_{\text{Id}}$) to 0 ($d_{0,i}$ is also 0).

The probabilistic latent variable $\boldsymbol{z}_{t}$ in each module is assumed to follow a multivariate Gaussian distribution with a diagonal covariance matrix, meaning $z_{t,i}$ and $z_{t,j}$ are independent ($i, j\in I_{\text{Cz}},I_{\text{Uz}},I_{\text{Az}},{{I_{\text{Pz}}, I}_{\text{Ez}}, I}_{\text{Iz}} \wedge i \neq j$). Here, the mean $\mu_{p,t,i}$ and sigma $\sigma_{p,t,i}$ (standard deviation) of the prior distribution $p(z_{t,i})$ in all modules are calculated as below:

$$\begin{aligned} p\left( z_{t,i} \right)= p\left( z_{t,i}|d_{t-1,j} \right)\mathcal{=N}\left( z_{t,i};\mu_{p,t,i},\sigma_{p,t,i} \right)\#(A.3) \end{aligned}$$

$$\begin{aligned} \mu_{p,t,i}=\tanh\left( \sum_{j} \omega_{ij}d_{t-1,j} \right)\#\left( A.4 \right) \end{aligned}$$

$$\begin{aligned} \sigma_{p,t,i}=\exp\left( \sum_{j} \omega_{ij}d_{t-1,j} \right)\#\left( A.5 \right) \end{aligned}$$

Here, prior distributions in each module are calculated from the previous RNN states of the same module, meaning $(i\in I_{\text{Cz}}\wedge j\in I_{\text{Cd}})\vee(i\in I_{\text{Uz}}\wedge j\in I_{\text{Ud}})\vee(i\in I_{\text{Az}}\wedge j\in I_{\text{Ad}})\vee(i\in I_{\text{Pz}}\wedge j\in I_{\text{Pd}})\vee(i\in I_{\text{Ez}}\wedge j\in I_{\text{Ed}})\vee(i\in I_{\text{Iz}}\wedge j\in I_{\text{Id}})$.

The posterior distribution in each module is calculated as,

$$\begin{aligned} q\left( z_{t,i}|\boldsymbol{e}_{t:T} \right)\mathcal{=N}\left( z_{t,i};\mu_{q,t,i},\sigma_{q,t,i} \right) \#\left( A.6 \right) \end{aligned}$$

$$\begin{aligned} \mu_{q,t,i}=\tanh\left( a_{\mu,t,i} \right)\#\left( A.7 \right) \end{aligned}$$

$$\begin{aligned} \sigma_{q,t,i}=\exp\left( a_{\sigma,t,i} \right)\#\left( A.8 \right) \end{aligned}$$

$$\begin{aligned} z_{t,i}=\mu_{q,t,i}+\sigma_{q,t,i}\times\epsilon\#\left( A.9 \right) \end{aligned}$$

Here, $\boldsymbol{a}_{t}$ is the adaptive internal state of the latent variables representing the posterior distributions. As described below, the adaptive variables $\boldsymbol{a}_{t}$ are determined by the error signals $\boldsymbol{e}_{t:T}$ propagated using the backpropagation through time (BPTT) algorithm ($T$ is the last timestep). The adaptive variables $\boldsymbol{a}_{t}$ (posterior distributions) are initialized by the corresponding initial internal states of the latent variables, which represent the prior distributions before posterior updating in the learning and online inferences. In equation (A.9), by sampling $\epsilon$ from $\mathcal{N}\left( 0,1 \right)$, the latent state $z_{t,i}$ is obtained.

Finally, mean predictions ${\hat{\boldsymbol{x}}}_{t}$ about proprioceptive, exteroceptive, and interoceptive sensations are individually generated from the corresponding modules, whereas the uncertainty (sigma) prediction ${\hat{\boldsymbol{\sigma}}}_{t}$ for each sensation was generated using the RNN states of the unexpected-uncertainty-cause module.

$$\hat{x}_{t,i}=\left\{ \begin{aligned} \tanh\left( \sum_{j\in I_{\text{Pd}}} \omega_{ij}d_{t,j} \right) \left( i\in I_{\text{Po}} \right) \\ \tanh\left( \sum_{j\in I_{\text{Ed}}} \omega_{ij}d_{t,j} \right) \left( i\in I_{\text{Eo}} \right) \\ \tanh\left( \sum_{j\in I_{\text{Id}}} \omega_{ij}d_{t,j} \right) \left( i\in I_{\text{Io}} \right) \\ \#\left( A.10 \right) \end{aligned} \right.$$

$$\begin{aligned} \hat{\sigma}_{x,t,i}=\exp\left( \sum_{j\in I_{\text{Ud}}} \omega_{ij}d_{t,j} \right) \left( i\in I_{\text{Po}},I_{\text{Eo}},I_{\text{Io}} \right) \#\left( A.11 \right) \end{aligned}$$

where $I_{\text{Po}}$, $I_{\text{Eo}}$, $I_{\text{Io}}$ are the index sets of the output units for the proprioceptive, exteroceptive, and interoceptive predictions, respectively.

***A.1.2. Learning***

The learning of the MBH-RNN was conducted offline through supervised learning using target sensory sequences $\boldsymbol{x}_{1:T}$ recorded in advance. Parameters, including synaptic weights $\boldsymbol{\omega}$ and adaptive variables $\boldsymbol{a}_{1:T}$ (posterior distributions), were updated based on the gradient descent method seeking to minimize variational free energy (VFE) over the time length of learning data $F_{learning}$.

$$\begin{aligned} F_{learning}=\sum_{t=1}^{T} \mathrm{VFE}_{t} \#\left( A.12 \right) \end{aligned}$$

$$\begin{aligned} \mathrm{VFE}_{t}=\underset{\text{Negative accuracy}}{\underbrace{-ln p\left( \boldsymbol{x}_{t} | \boldsymbol{d}_{t} \right)}}\boldsymbol{+}\sum_{m} \underset{\text{Complexity}}{\underbrace{D_{KL}\left[ q\boldsymbol{(}\boldsymbol{z}_{t}^{\left( m \right)}\boldsymbol{|}\boldsymbol{e}_{t:T}\boldsymbol{)||}p\boldsymbol{(}\boldsymbol{z}_{t}^{\left( m \right)}\boldsymbol{|}\boldsymbol{d}_{t-1}^{\left( m \right)}\boldsymbol{)} \right]}}\#\left( A.13 \right) \end{aligned}$$

where $m$ denotes a network module. The first term (negative accuracy term) of the VFE describes the negative log-likelihood (NLL) or precision-weighted sensory prediction error. For simplicity, we assume that each sensation is independent and follows a Gaussian distribution.

$$\begin{aligned} \text{Negative accuracy}=\sum_{i\in I_{\text{Po}}\bigvee I_{\text{Eo}}\bigvee I_{\text{Io}}} \left( \frac{\left( x_{t,i}-\hat{x}_{t,i} \right)^{2}}{2\hat{\sigma}_{x,t,i}^{2}}+\frac{\ln\left( 2\pi\hat{\sigma}_{x,t,i}^{2} \right)}{2} \right)\#\left( A.14 \right) \end{aligned}$$

We divided the negative accuracy term by the dimensions of each exteroceptive and proprioceptive sensation as follows:

The second term (complexity term) of the VFE describes the Kullback-Leibler divergence (KLD) between the posteriors $\boldsymbol{q(}\boldsymbol{z}_{t}^{\left( m \right)}\boldsymbol{|}\boldsymbol{e}_{t:T}\boldsymbol{)}$ and priors $\boldsymbol{p(}\boldsymbol{z}_{t}^{\left( m \right)}\boldsymbol{|}\boldsymbol{d}_{t-1}^{\left( m \right)}\boldsymbol{)}$. Assuming that the prior and posterior distributions follow a multivariate Gaussian distribution with a diagonal covariance matrix, as described above, KLD is computed as follows:

$$\begin{aligned} \text{Complexity}=\sum_{i\in I_{\text{Mz}}} \left( \ln\frac{\sigma_{p,t,i}}{\sigma_{q,t,i}}+\frac{\left( \mu_{p,t,i}-\mu_{q,t,i} \right)^{2}+\left( \sigma_{q,t,i} \right)^{2}}{2\left( \sigma_{p,t,i} \right)^{2}}-\frac{1}{2} \right)\#\left( A.15 \right) \end{aligned}$$

$I_{\text{Mz}}$ is the index set of probabilistic latent variables in the corresponding module $m$. We divided the complexity term by the dimensions of the latent variables for each module.

The partial derivative of $F_{learning}$ with respect to each parameter can be determined using the BPTT algorithm. We used the rectified Adam method for parameter updates via gradient descent.

***A.1.3. Online perceptual inference and allostatic goal modulation***

In the online inference and goal modulation phases after learning, the MBH-RNN performs posterior updates within timesteps from $t_{c}-{win}_{p}+1$ to $t_{c}+{win}_{f}$ at the current network timestep $t_{c}$ (${win}_{p}$ and ${win}_{f}$are the lengths of the past and future time windows, respectively) (Fig. 4). The cost function in the past time window $F_{past}$ is the accumulated VFE of the past (VFEP), based on the observed sensations $\boldsymbol{x}_{t_{c}-{win}_{p}+1:t_{c}}$.

$$\begin{aligned} F_{past}=\sum_{t=t_{c}-{win}_{p}+1}^{t_{c}} \mathrm{VFEP}_{t}\#\left( A.16 \right) \end{aligned}$$

$$\begin{aligned} \mathrm{VFEP}_{t}=\underset{\text{Negative accuracy}}{\underbrace{-ln p\left( \boldsymbol{x}_{t} | \boldsymbol{d}_{t} \right)}}\boldsymbol{+}W\sum_{m} \underset{\text{Complexity}}{\underbrace{D_{KL}\left[ q\boldsymbol{(}\boldsymbol{z}_{t}^{\left( m \right)}\boldsymbol{|}\boldsymbol{e}_{t:t_{c}+{win}_{f}}\boldsymbol{)||}p\boldsymbol{(}\boldsymbol{z}_{t}^{\left( m \right)}\boldsymbol{|}\boldsymbol{d}_{t-1}^{\left( m \right)}\boldsymbol{)} \right]}}\#\left( A.17 \right) \end{aligned}$$

The difference from $F_{learning}$ is that we introduce a hyperparameter called the meta-prior $W,$ which controls the balance between the negative accuracy term and the divergence between the posterior and prior at each module.

For future predictive processing, we extend the VFE to the future time window while considering that sensations in the future are unknown variables. Specifically, we derived the VFE of the future (VFEF) by evaluating VFE under the expectation of predictions about future sensations. This changed the NLL term of the VFE to the predicted conditional (Shannon) entropy of sensation. The cost function in the future time window $F_{future}$ is the VFEF accumulated over the future time window.

$$\begin{aligned} F_{future}=\sum_{t=t_{c}+1}^{t_{c}+{win}_{f}} \mathrm{VFEF}_{t}\#\left( A.18 \right) \end{aligned}$$

$$\begin{aligned} \mathrm{VFEF}_{t}=\underset{\text{Predicted sensory entropy}}{\underbrace{\mathcal{H}\left[ p\left( \boldsymbol{x}_{t}\boldsymbol{|}\boldsymbol{d}_{t} \right) \right]}}\boldsymbol{+}W\sum_{m} \underset{\text{Predicted complexity}}{\underbrace{D_{KL}\left[ q\boldsymbol{(}\boldsymbol{z}_{t}^{\left( m \right)}\boldsymbol{|}\boldsymbol{e}_{t:t_{c}+{win}_{f}}\boldsymbol{)||}p\boldsymbol{(}\boldsymbol{z}_{t}^{\left( m \right)}\boldsymbol{|}\boldsymbol{d}_{t-1}^{\left( m \right)}\boldsymbol{)} \right]}}\#\left( A.19 \right) \end{aligned}$$

$$\begin{aligned} \text{Predicted sensory entropy}=\sum_{i\in I_{\text{Po}}\bigvee I_{\text{Eo}}\bigvee I_{\text{Io}}} \left( \frac{1}{2}+\frac{\ln\left( 2\pi\hat{\sigma}_{x,t,i}^{2} \right)}{2} \right)\#\left( A.20 \right) \end{aligned}$$

This derivation of VFEF allows for the formalization of allostatic processing to avoid an increase in entropy (uncertainty).

Taken together, the MBH-RNN with fixed synaptic weights repeats prediction generations and updates of adaptive variables $\boldsymbol{a}_{t_{c}-{win}_{p}+1:t_{c}+{win}_{f}}$ (posterior distributions) within timesteps from $t_{c}-{win}_{p}+1$ to $t_{c}+{win}_{f}$ to minimize the accumulated free energy from the past to future $F_{allostasis}$.

$$\begin{aligned} F_{allostasis}=F_{past}+F_{future}\#\left( A.21 \right) \end{aligned}$$

In the resting state experiment, we assume that the interoceptive state was constantly 0.0 ($x_{t, int}=0.0$ for all $t$) while exteroceptive and proprioceptive prediction errors were not considered ($\boldsymbol{x}_{t,ext}={\hat{\boldsymbol{x}}}_{t,ext}$ and $\boldsymbol{x}_{t,pro}={\hat{\boldsymbol{x}}}_{t,pro}$for all $t$).

***A.1.4. Hyper-parameter setting***

The hyperparameter settings were similar to those used in a previous study (Idei, Tani, Ogata, & Yamashita, 2024a) The dimensions of the latent variables $\boldsymbol{z}$  and RNN units $\boldsymbol{d}$ in the proprioceptive, exteroceptive, interoceptive, multimodal-associative, unexpected-uncertainty-cause, and higher cognitive modules were $N_{\mathrm{Pz}}=2, N_{\mathrm{Ez}}=2, N_{\mathrm{Iz}}=1, N_{\mathrm{Az}}=8,N_{\mathrm{Uz}}=1,N_{\mathrm{Cz}}=3$ (for latent variables) and ${N_{\mathrm{Pd}}=10,{N_{\mathrm{Ed}}=20, N}_{\mathrm{Id}}=10, {N_{\mathrm{Ad}}=20, N}_{\mathrm{Ud}}=10, N}_{\mathrm{Cd}}=10$ (for RNN units), respectively. For the time constant $\tau$ of RNN units, we set the time constant for each of the lower perceptual modules as $\tau_{\mathrm{Pd}}=\tau_{\mathrm{Ed}}=\tau_{\mathrm{Id}}=2 \mathrm{or} 4$ (for each of the half the number of RNN units). Subsequently, time constants in the multimodal-associative module and unexpected-uncertainty-cause module were set as $\tau_{\mathrm{Ad}}=\tau_{\mathrm{Ud}}=4 \mathrm{or} 8$ (for each of the half RNN units), while those in the higher cognitive module were set as $\tau_{\mathrm{Cd}}=8 \mathrm{or} 16$ (for each of the half RNN units). Accordingly, higher-level modules have a larger time constant (slower neural dynamics) than lower-level modules, which introduces a temporal hierarchy. The synaptic weights were initialized with random values using the Xavier method. The biases of the RNN units were initialized with and fixed to random values following a Gaussian distribution $\mathcal{N}(0,10)$. Parameters including synaptic weights and adaptive variables were updated using the rectified Adam optimizer (learning rate ${lr}_{learning}=0.01$, $\beta_{1}=0.9$, $\beta_{2}=0.999$).

***A.2. Supplementary figure***

**Supplementary Fig. S1: *Effects of changing the length of past or future time windows on mentally simulated behavior***

**(a)** Effects of changing the length of the previous time window ${win}_{p}$ on the occurrence rates of rest, wandering, and moving distance. Values on the horizontal axis are expressed as multiples of the baseline values (${win}_{p}=10$). The deep-being mode (increase in resting) was not induced by alterations in the length of the past time window; however, a decreased length of the previous time window led to a decrease in resting (increase in wandering). **(b)** Effects of changing the length of the future time window ${win}_{f}$ on the occurrence rates of resting, wandering, and moving distances. The values on the horizontal axis are expressed as multiples of the baseline values (${win}_{f}=200$). Alterations in the length of the future time window did not strongly influence mentally simulated behavior.

**Supplementary Fig. S1 Alt-text:**

Effects of varying the lengths of past and future time windows on mentally simulated behavior. Panel (a) shows that shortening the past time window reduces resting behavior and increases wandering, whereas increasing the past time window has little effect and does not induce the deep-being mode. Panel (b) shows that changes in the future time window have minimal influence on the occurrence of resting and wandering states or on simulated movement distance.
